# Optimal BR signalling is required for adequate cell wall orientation in the *Arabidopsis* root meristem

**DOI:** 10.1242/dev.199504

**Published:** 2021-11-10

**Authors:** Zhenni Li, Ayala Sela, Yulia Fridman, Lucía Garstka, Herman Höfte, Sigal Savaldi-Goldstein, Sebastian Wolf

**Affiliations:** 1 Department of Cell Biology, Centre for Organismal Studies Heidelberg, Heidelberg University, Im Neuenheimer Feld 230, 69120 Heidelberg, Germany; 2 Plant Biology Laboratory, Technion-Israel Institute of Technology, Haifa 3200003, Israel; 3 Department of Development, Signalling, and Modelling, Institut Jean-Pierre Bourgin, INRA, AgroParisTech, CNRS, Université Paris-Saclay, 78000 Versailles, France; 4 Department of Plant Biochemistry, Centre for Plant Molecular Biology (ZMBP), Eberhard Karls University, D-72076 Tübingen, Germany

**Keywords:** Brassinosteroids, Cell wall, Cell division, *Arabidopsis*, Root, Meristem

## Abstract

Plant brassinosteroid hormones (BRs) regulate growth in part through altering the properties of the cell wall, the extracellular matrix of plant cells. Conversely, feedback signalling from the wall connects the state of cell wall homeostasis to the BR receptor complex and modulates BR activity. Here, we report that both pectin-triggered cell wall signalling and impaired BR signalling result in altered cell wall orientation in the *Arabidopsis* root meristem. Furthermore, both depletion of endogenous BRs and exogenous supply of BRs triggered these defects. Cell wall signalling-induced alterations in the orientation of newly placed walls appear to occur late during cytokinesis, after initial positioning of the cortical division zone. Tissue-specific perturbations of BR signalling revealed that the cellular malfunction is unrelated to previously described whole organ growth defects. Thus, tissue type separates the pleiotropic effects of cell wall/BR signals and highlights their importance during cell wall placement.

## Abstract (precis)

**Summary:** Both increased and reduced BR signalling strength results in altered cell wall orientation in the *Arabidopsis* root, uncoupled from whole-root growth control.

## INTRODUCTION

The cell wall, a carbohydrate-rich structure surrounding all plant cells, fulfils numerous essential functions in growth and development; it provides mechanical support, controls cellular adhesion and forms the interface with the environment (Cosgrove, 2016; Lampugnani et al., 2018; Voxeur and Höfte, 2016). Notably, as the cell wall prevents cell migration, tight control over the plane of cell wall deposition during cytokinesis is often assumed to be important for plant patterning and morphogenesis. However, whether there is a direct relationship between cell shape, in part controlled by cell division orientations, and organ shape is not clear (Beemster et al., 2003; Kaplan and Hagemann, 1991; Torres-Ruiz and Jurgens, 1994; Traas et al., 1995). During cytokinesis, the microtubules (MT) of the phragmoplast guide secretory traffic towards the cell plate, a radially expanding disk of membrane-engulfed cell wall material that increases in diameter until it fuses with the parental walls to conclude cell division (Livanos and Müller, 2019). Cell division plane orientation is pre-determined before the cell plate actually develops. Immediately before prophase, the MT network forms a transient ring structure known as preprophase band (PPB) at the periphery of the cell, marking the future fusion site of the cell plate and the parental wall during cytokinesis (Livanos and Müller, 2019; Rasmussen and Bellinger, 2018; Rasmussen et al., 2013; Smith, 2001). This site, called the cortical division zone (CDZ) persists throughout mitosis and is populated by proteins that guide the expanding cell plate towards the parental wall. Although PPBs are at least partially dispensable for oriented cell division (Schaefer et al., 2017; Zhang et al., 2016), mutants in CDZ components such as TAN1 and POK1,2 show severely altered division angles, suggesting that these factors are important for phragmoplast guidance in the absence of these factors (Lipka et al., 2014; Stöckle et al., 2016; Walker et al., 2007). In light of the central role of cell wall biosynthesis during cytokinesis (Gu et al., 2016; Miart et al., 2014; Zuo et al., 2000) and a growing list of cell wall-mediated feedback signalling modules affecting a wide range of biological processes (Wolf, 2017), it is conceivable that cell wall state during mitosis/cytokinesis has to be monitored by cell wall surveillance systems (Rui and Dinneny, 2020; Vaahtera et al., 2019; Voxeur and Höfte, 2016).

Growth itself poses a threat to cell wall integrity and composition and physical properties of the cell wall have to be tightly monitored to ensure cell wall homeostasis and coordinated growth. For example, a compensatory response to cell wall challenge is mediated by RECEPTOR-LIKE PROTEIN 44 (RLP44), which is able to interact with the brassinosteroid (BR) receptor BRI1 (Holzwart et al., 2018) and its co-receptor BRI1-ASSOCIATED KINASE1 (BAK1) (Wolf et al., 2014) to promote BR signalling. More specifically, the degree of methylesterification (DM) of homogalacturonan (HG), a pectin component of the cell wall, has a profound impact on cell wall structure and mechanical properties: once secreted into the wall network, HG can be de-methylesterified by pectin methylesterases (PMEs), ubiquitous plant enzymes which are themselves regulated by PME inhibitor proteins (PMEIs). Reduction of PME activity through overexpression of *PECTIN METHYLESTERASE INHIBITOR 5* (*PMEI5*) activates BR hormone signalling via RLP44, which in turn leads to a complex directional growth phenotype including organ fusion and root waving (Wolf et al., 2012, 2014).

BR-deficient mutants and plants treated with BR biosynthesis inhibitors display reduced longitudinal meristem size (Cole et al., 2014; González-García et al., 2011; Hacham et al., 2011; Vragović et al., 2015), but show an increased number of formative (periclinal) divisions, resulting in supernumerary cell files in root tissues (Holzwart et al., 2018; Kang et al., 2017). In addition to the main receptor BRI1, BRs are also perceived by two close paralogs of BRI1, BRL1 and BRL3 (Caño-Delgado et al., 2004; Zhou et al., 2004). *brl1* and *brl3* single and double mutants do not show growth defects and *bri1 brl1 brl3* triple mutant (*bri1*-triple from hereon) resembles *bri1*. However, the absence of *brl1* and *brl3* enhances the vascular defects of *bri1* (Caño-Delgado et al., 2004; Holzwart et al., 2018; Kang et al., 2017). BR signalling has context- and cell type-specific roles in plant growth and development (Ackerman-Lavert and Savaldi-Goldstein, 2019; Nolan et al., 2019; Planas-Riverola et al., 2019; Singh and Savaldi-Goldstein, 2015), involving cell autonomous and non-cell autonomous effects on root length and on the number of anticlinal and periclinal divisions in the meristem (Hacham et al., 2011; Vragović et al., 2015; Kang et al., 2017; Graeff et al., 2020).

Here, we dissected the phenotype induced by PMEI5-mediated reduction in PME activity on the root apical meristem and revealed that pectin-triggered cell wall signalling leads to orientation defects of newly placed walls, which are dependent on BR signalling activation. These defects are dependent on BR signalling activation, but independent of organ-level growth phenotypes, and coincide with aberrant localisation of the CDZ component POK1. Conversely, reduced BR signalling in receptor and biosynthetic mutants leads to cell wall orientation defects similar to those observed in PMEIox, but unrelated to the enhanced formative division phenotypes. These cell wall orientation defects can also be genetically separated from BR functions in root growth. Thus, we reveal a role for cell wall and BR signalling in controlling cell wall orientation.

## RESULTS

### Pectin-triggered cell wall signalling leads to cell division defects that are RLP44 and BRI1 dependent

**Fig. 1.**
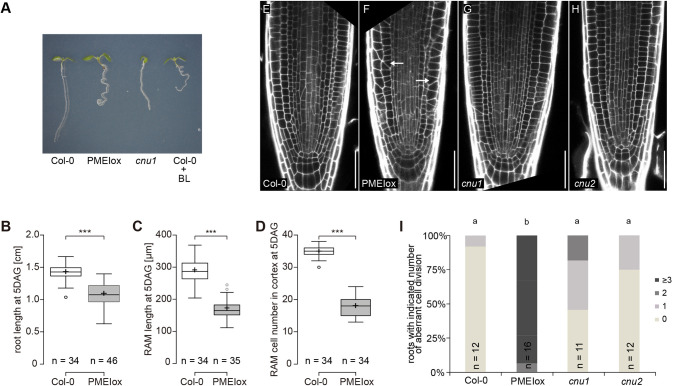
**Cell wall signalling triggered by ubiquitous PMEI5 expression in PMEIox alters root growth and cell wall orientation.** (A) PMEIox seedlings have a root waving phenotype caused by enhanced BR signalling: 5-day-old seedlings of Col-0, PMEIox, the PMEIox *bri1* suppressor mutant *cnu1*, and Col-0 seedlings grown on plates containing 5 nM brassinolide (BL) are shown. (B-D) PMEIox plants show reduced root length (B), RAM length (C) and RAM cell number (D) 5 DAG. ***P<0.001 (Mann–Whitney *U*-test). Boxes indicate median, upper and lower quartile, whiskers indicate minimum and maximum except outliers beyond 1.5× interquartile range, which are indicated as dots. Cross indicates mean. (E-H) PMEIox plants show oblique cell walls in the root apical meristem, dependent on BRI1 and RLP44. Cell division defects (compared with Col-0; E) in PMEIox (F, arrows) are reduced in the PMEIox suppressor mutants *cnu1*, carrying a mutation in BRI1 (G), and *cnu2*, carrying a mutation in RLP44 (H). Cells walls of the root apical meristems are visualised using mPS-PI staining (Truernit et al., 2008). Scale bars: 50 µm. (I) Quantification of the fraction of roots with the indicated number of oblique transversal walls in cortex cells from confocal section as in E-H. Letters indicate statistically significant differences according to Dunn's test after Kruskal–Wallis analysis.

We have previously demonstrated that cell wall signalling connects the state of the cell wall to the BR signalling pathway (Wolf et al., 2012). When de-methylesterified pectin becomes limiting, such as in PMEIox plants overexpressing *PMEI5*, elevated BR signalling counteracts loss of cell wall integrity and leads to BRI1-dependent macroscopic growth defects such as reduced root length and root waving (Wolf et al., 2012; Fig. 1A,B). In agreement with this, external application of brassinolide (BL) also results in root waving (Lanza et al., 2012; Wolf et al., 2012) (Fig. 1A). To shed light on the cellular phenotype of PMEIox roots and to assess whether root waving is associated with meristematic defects, we analysed PMEIox root tips 5 days after germination (DAG) and found that the reduced root length of PMEIox was accompanied by a reduction in size and cell number of the root apical meristem (RAM) (Fig. 1C,D). Interestingly, we also revealed that, in contrast to the stereotypical pattern of cellular morphology and tissue organisation in wild-type root tips (Dolan et al., 1993) (Fig. 1E), PMEIox roots displayed a substantial amount of obliquely orientated transverse cell walls apparent in epidermis, cortex and endodermis (Fig. 1F). We next asked whether these cell wall orientation defects in PMEIox are also the result of elevated BR signalling mediated by RLP44 (Wolf et al., 2014). Consistent with this hypothesis, PMEIox suppressor mutants with lesions in *BRI1* (*cnu1*; Wolf et al., 2012) and *RLP44* (*cnu2*; Wolf et al., 2014), respectively, showed to a large extent normal cross wall orientation (Fig. 1G-I). Thus, cell wall orientation defects in PMEIox are mediated by RLP44 and BRI1, similar to the other macroscopic PMEIox phenotypes, such a root waving and organ fusion (Fig. 1A) (Wolf et al., 2012, 2014). Moreover, expression of a GFP-tagged version of RLP44 under control of the CaMV 35S promoter (RLP44-GFP) in *cnu2* resulted in a significant increase of oblique cell walls, indicating complementation (Fig. S1). Together, these results suggest that cell wall signalling-triggered elevation of BR signalling is causative for the oblique cell wall phenotype in PMEIox.

### Cell wall perturbation in diverse cell types separates aberrant cell division, root waviness and root growth

**Fig. 2.**
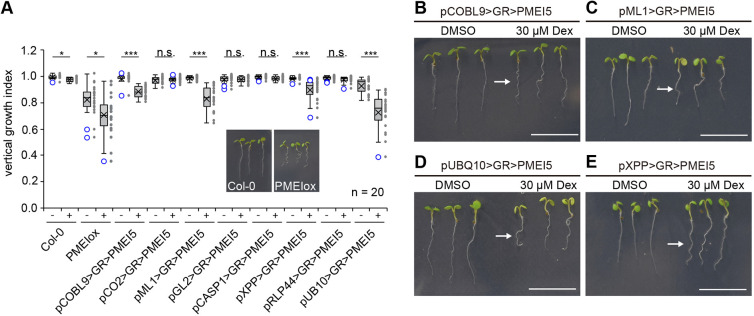
**Cell wall perturbation in diverse cell types can lead to similar organ-level responses.** (A) Quantification of the vertical growth index (distance from hypocotyl junction to tip of the root divided by root length) in Col-0, PMEIox and inducible tissue-specific expression lines in the absence and presence of 30 µM dexamethasone (DEX). (B-E) Induction of PMEIox trans-activation in hair cells (B), epidermal cells (C), ubiquitously (D) and in meristematic cortex cells (E) leads to root waving. Plants were germinated and grown on plates containing 30 μM DEX or an equal volume of DMSO for 5 days. Scale bars: 1 cm. *P<0.05, ***P<0.001 (Mann–Whitney *U*-test). n.s., not significant. Boxes indicate median, upper and lower quartile, whiskers indicate minimum and maximum except outliers beyond 1.5× interquartile range, which are indicated as blue circles. Cross indicates mean. Individual data points (*n*=20) are shown to the right of the box diagrams.

**Fig. 3.**
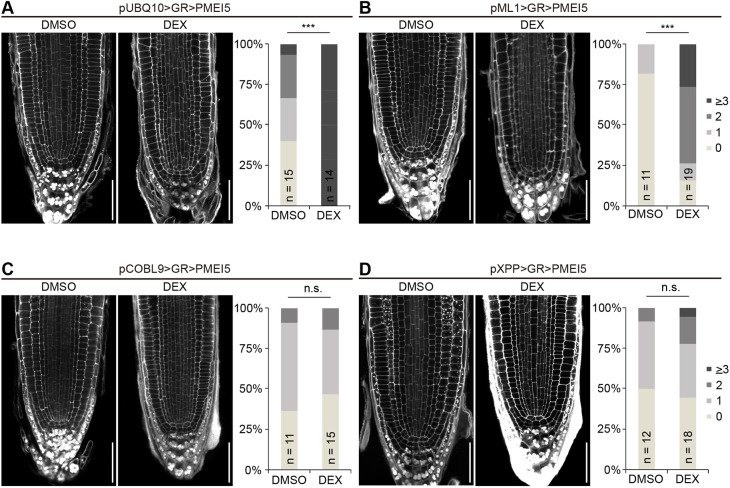
**Tissue-specific expression of PMEIox reveals that root waving is independent from cell wall orientation defects.** (A) Ubiquitous, pUBQ10-driven trans-activation of *PMEI5* recapitulates the PMEIox cell wall phenotype. (B-D) In lines with tissue-specific PMEI5 expression in epidermis, hair cells or XPP cells, cell wall orientation shows varying degrees of cell wall orientation defects. Scale bars: 50 µm. Cell walls are counterstained with mPS-PI. Bar diagrams denote fraction of roots with the indicated number of oblique transversal walls in epidermis cells (A-C) or all cells (D) from confocal sections. ***P<0.001 (Mann–Whitney *U*-test). n.s., not significant.

**Fig. 4.**
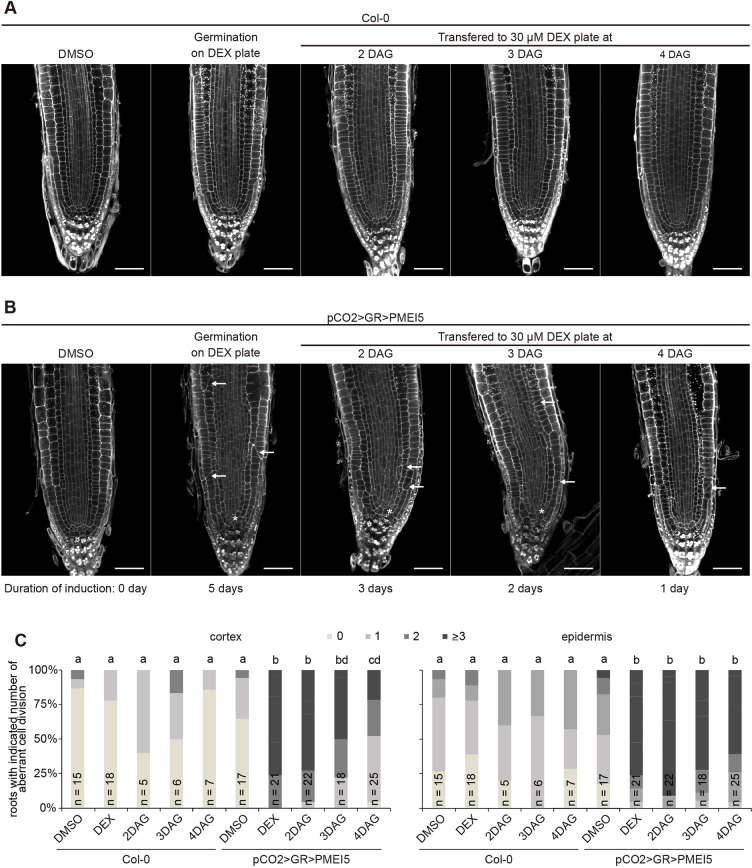
**Cell wall orientation defects allow unaltered organ level growth.** (A,B) Induction of *PMEI5* expression in meristematic cortex cells leads to oblique cell walls in cortex and epidermis (arrows) and a disrupted stem cell region (asterisk). Plants were imaged at 5 DAG on DEX-containing medium or after germination on DMSO-containing plates and transferred to induction medium at the indicated time points. (C) Quantification of the fraction of roots with the indicated number of oblique transversal walls in cortex cells from confocal sections. Letters indicate statistically significant differences according to Dunn's test after Kruskal–Wallis analysis.

Thus far, our results indicate that pectin-triggered cell wall signalling leads to root waving, root growth inhibition and aberrant wall orientation phenotypes, which are all BRI1-dependent. These pleiotropic effects could be all linked or could result from unrelated processes. As BR signalling is context-dependent, we employed a cell type-specific expression system to alter cell wall properties locally and followed the phenotypic consequences at the organ, tissue and cellular level. We selected a number of tissue-specific promoters to drive expression of the chimeric transcription factor GR-LhG4 (Craft et al., 2005; Moore et al., 1998; Schürholz et al., 2018) in the epidermis, cortex, endodermis or xylem pole pericycle cells, complemented by ubiquitous expression (Fig. S2). GR-LhG4, in turn, triggers transcription of *PMEI5* under control of the synthetic pOp promoter in the presence of dexamethasone (DEX). Quantitative reverse transcription PCR (QRT-PCR) revealed that, in all lines, PMEI5 was upregulated by DEX treatment, even though absolute levels differed substantially between lines (Fig. S3). These differences might, at least in part, depend on varying strength and cell type-specificity of the respective promoters. To assess root waving of PMEI5-expressing lines, we determined the vertical growth index of 6-day-old seedlings grown on medium containing either DEX or an equal amount of DMSO (Fig. 2A). Expression of *PMEI5* in hair cells, driven by the *pCOBL9* promoter (pCOBL9>GR>PMEI5; Fig. 2A,B), or in all epidermal cells (pML1>GR>PMEI5; Fig. 2A,C) was sufficient to trigger root waving, similar to ubiquitous expression (pUBQ10>GR>PMEI5; Fig. 2A,D). Interestingly, expression in the xylem pole pericycle (XPP) cells of the stele (pXPP>GR>PMEI5) also led to a root waving phenotype (Fig. 2A,E). This suggests that cell wall-induced BR signalling in these cells causes organ-level responses, with the caveat that PMEI5 might be mobile in the cell wall. We then assessed the occurrence of oblique cell walls in the trans-activation lines. Notably, tissue-specific expression lines that displayed root waving, namely pUBQ10>GR>PMEI5, pML1>GR>PMEI5, pCOBL9>GR>PMEI5 and pXPP>GR>PMEI5, differed in their behaviour with respect to the cell wall orientation phenotype (Fig. 3). Whereas ubiquitous *PMEI5* expression (Fig. 3A) and expression under control of the *ML1* promoter (Fig. 3B) resulted in significant increase of aberrant epidermal cell wall placement, plants with hair cell (Fig. 3C) and pericycle expression of *PMEI5* (Fig. 3D) were unaffected. This suggests that PMEI5-induced root waving is at least partially independent from meristematic cell wall orientation defects. Interestingly, trans-activation of *PMEI5* in cortex cells (pCO2>GR>PMEI5), which did not lead to root waving (Fig. 2A), triggered PMEIox-like oblique cell walls cell autonomously in cortex cells and in neighbouring cell types such as epidermal cells (Fig. 4). Introducing a fluorescent reporter under control of the pOp6 promoter suggested that trans-activation in the pCO2>GR>PMEI5 line is indeed restricted to cortex cells (Fig. S4). However, it cannot be excluded that the observed non-cell autonomous effects were conferred by PMEI5 mobility in the apoplastic space. As the addition of fluorescent proteins or any other tags tested renders PMEI5 non-functional, this could not be experimentally addressed. In addition to the cell wall orientation defects, *PMEI5* expression in cortex cells resulted in a disrupted organisation of the stem cell niche (Fig. 4B) and a reduction of meristematic cell number (Fig. S5A). In marked contrast to PMEIox (Fig. 1), the effect on cell wall orientation in pCO2>GR>PMEI5 plants was not accompanied by a significant change of root length or RAM size compared with the uninduced control (Fig. S5B,C). Taken together, these results reveal that triggering cell wall modification in either the epidermis or in XPP cells of the stele can lead to similar organ level responses and confirm that PMEI5-induced cell wall orientation alterations are independent of directional growth processes. In addition, meristematic cell wall orientation defects in pCO2>GR>PMEI5 did not affect organ-level growth.

### Reduced BR signalling leads to wall orientation defects that can be separated from root growth

**Fig. 5.**
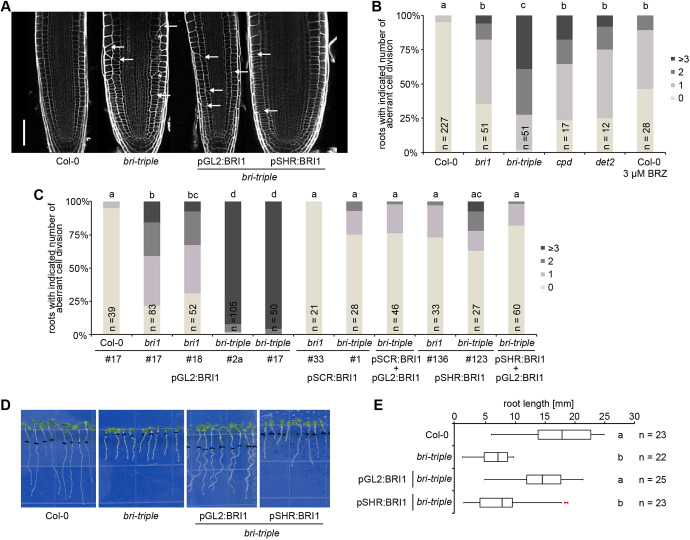
**BR signalling is required for the maintenance of cell wall orientation.** (A) PI-stained meristems of Col-0, *bri1*-triple mutants and two *bri1*-triple lines expressing BRI1 from cells of the epidermis (pGL2:BRI1) or stele (pSHR:BRI1). Note oblique transversal walls in epidermis, cortex and endodermis (arrows), as well as random perturbation of cell files (asterisk). (B) Quantification of the fraction of cortex cells with oblique transversal walls in cortex cells from confocal section in A for the indicated genotypes and wild-type roots in which BRs were depleted by treatment with BRZ. Numbers in the *x*-axis indicate independent transgenic lines. Letters indicate statistically significant differences according to Dunn's test after Kruskal–Wallis analysis. (C) Quantification of the fraction of cortex cells with altered cell wall orientation of lines expressing BRI1 from the epidermis (pGL2-BRI1), endodermis (pSCR-BRI1) and stele (pSHR-BRI1) as well as their combinations in various backgrounds. Letters indicate statistically significant differences according to Dunn's test after Kruskal–Wallis analysis. (D) Growth phenotype of 7-day-old Col-0, *bri1*-triple and *bri1*-triple plants expressing BRI1 from cells of the epidermis (pGL2:BRI1) or stele (pSHR:BRI1). (E) Quantification of root length as depicted in D. *n*=21-25. Letters indicate statistically significant difference after one-way ANOVA followed by Tukey's post-hoc test. Boxes indicate median, upper and lower quartile, whiskers indicate minimum and maximum except outlier beyond 1.5× interquartile range, which is indicated as red cross.

We next characterised the cross-wall orientation in lines with reduced BR signalling and observed aberrant wall angles in *bri1* that were enhanced in *bri1*-triple mutants. The occurrence of oblique transversal walls within the cell files of the root meristem resulted in random perturbations (Fig. 5A, asterisk), differing from the formative divisions previously observed in these mutants that result in additional cell files (Holzwart et al., 2018; Kang et al., 2017). Quantification of aberrant cell walls in cortex cells revealed that the majority of *bri1* meristems had at least one oblique cortex cross wall per median confocal section, a phenotype which was enhanced in *bri1*-triple mutants (Fig. 5B). We recently reported that some functions of BRI1 are independent of classical BR signalling outputs (Holzwart et al., 2018, 2020). However, cell wall orientation defects in *bri1* mutants appear to be caused by reduced canonical BRI1/ligand dependent signalling, as the biosynthetic mutants *cpd* (Szekeres et al., 1996) and *det2* (Chory et al., 1991), as well as plants in which endogenous BRs were depleted by the application of brassinazole (BRZ) (Asami et al., 2000), displayed phenotypes comparable with *bri1* mutants (Fig. 5B). To address whether the impact of BR activity on the cell wall orientation is tissue-specific, we used a previously reported collection of lines conferring tissue-specific BRI1 expression in *bri1* and *bri1*-triple mutant backgrounds (Fridman et al., 2014; Hacham et al., 2011; Vragović et al., 2015). In contrast to expression of BRI1 in non-hair cells (pGL2-BRI1), expression of BRI1 from the endodermis (pSCR-BRI1) or stele (pSHR-BRI1) largely rescued cell wall orientation in the cortex of *bri1* and *bri1*-triple mutants (Fig. 5C). Interestingly, the extent of the effect of BRI1 on the aberrant wall angles was not linked to its effect on meristem size and root length (Hacham et al., 2011; Vragović et al., 2015; Fig. 5D,E; Fig. S6). Specifically, pGL2-BRI1 roots are longer than pSCR-BRI1 and pSHR-BRI1 and can have almost wild-type length, in both *bri1* and *bri1*-triple backgrounds (Hacham et al., 2011; Fig. 5D,E), despite the occurrence of aberrant cell walls (Fig. 5C). BRI1 activity in non-hair cells limits cell elongation in the elongation zone of the root, unless BRI1 in the neighbouring hair cells is also present (Fridman et al., 2014). High BRI1 in non-hair cells results in waviness in a mutant and wild-type background (Fridman et al., 2014; Fig. 5D). Thus, aberrant wall orientation is unlinked from root waving. Taken together, as with PMEIox, the control of cell wall orientation is separated from other growth processes controlled by BRI1. In addition, both enhanced BR signalling (PMEI5 expression) and reduced BR signalling (BR loss-of-function mutants) lead to cell wall orientation defects.

**Fig. 6.**
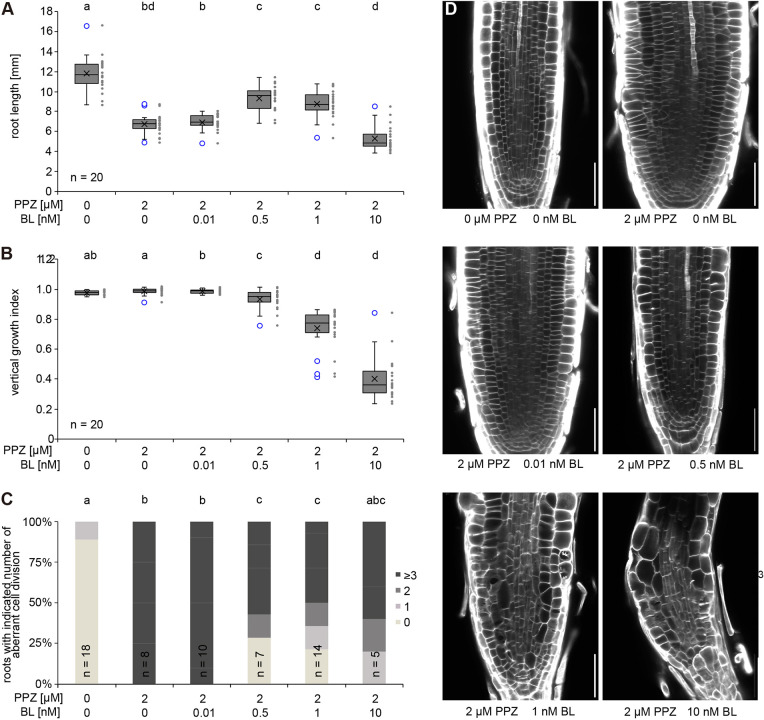
**Optimal BR signalling strength is required for normal cell wall orientation.** (A) Root length of Col-0 seedling untreated or grown in the presence of the BR biosynthesis inhibitor PPZ and the indicated concentration of epi-BL. Letters indicate statistically significant difference according to Tukey's HSD test after one-way ANOVA, *n*=20. (B) Vertical growth index of the same plants as in A. Letters indicate statistically significant differences according to Dunn's test after Kruskal–Wallis analysis, *n*=20. Boxes in A and B indicate median, upper and lower quartile, whiskers indicate minimum and maximum except outliers beyond 1.5× interquartile range, which are indicated as blue circles. Cross indicates mean. Individual data point are shown to the right of the box diagrams. (C) Quantification of the fraction of roots with the indicated number of oblique transversal walls in cortex cells from confocal sections in D. Letters indicate statistically significant differences according to Dunn's test after Kruskal–Wallis analysis. (D) Representative images of roots analysed in A-C. Cell walls were stained with SCRI 2200 in ClearSee (see Materials and Methods). Scale bars: 50 μm.

To independently test whether both growth-enhancing and growth-inhibiting doses of BRs affect the orientation of the new cell wall, plants were grown on medium containing the BR biosynthesis inhibitor propiconazole (PPZ) and supplemented with increasing concentrations of epi-BL. Treatment with PPZ inhibited root growth, which could be partially compensated by the addition of 0.5 nM epi-BL, without induction of root waving (Fig. 6A,B). Addition of 1 nM or 10 nM epi-BL to the PPZ-containing medium led to root waving and a reduction of root growth (Fig. 6A,B), demonstrating that these concentrations enhanced BR signalling. Both reduced and enhanced BR signalling strength resulted in aberrant cell wall orientation in root meristems (Fig. 6C,D), supporting our genetic evidence. Moreover, the cell wall orientation defects in pCO2>GR>PMEI5 roots (Fig. 4) were attenuated by loss of RLP44 function, and undetectable in a cross of the pCO2>GR>PMEI5 line with the hypomorphic *BRI1* mutant *bri1^cnu4^* (Holzwart et al., 2020) (Fig. S7). *The bri1^cnu4^* mutant showed a significantly increased frequency of aberrant cell wall orientations, similar to pCO2>GR>PMEI5 (Fig. S7) and consistent with our findings on other BR loss-of-function mutants (Fig. 5). However, pCO2>GR>PMEI5 (*bri1^cnu4^*) roots showed wild type-like cell wall orientation (Fig. S7), suggesting that the reduction of BR signalling strength caused by the BRI1 mutation can counteract the PMEI5-induced activation of BR signalling. Together, optimal BR signalling strength is required for normal cell wall orientation in the *Arabidopsis* root.

### Cell wall perturbation by PMEIox leads to cytokinesis defects after specification of the CDZ

**Fig. 7.**
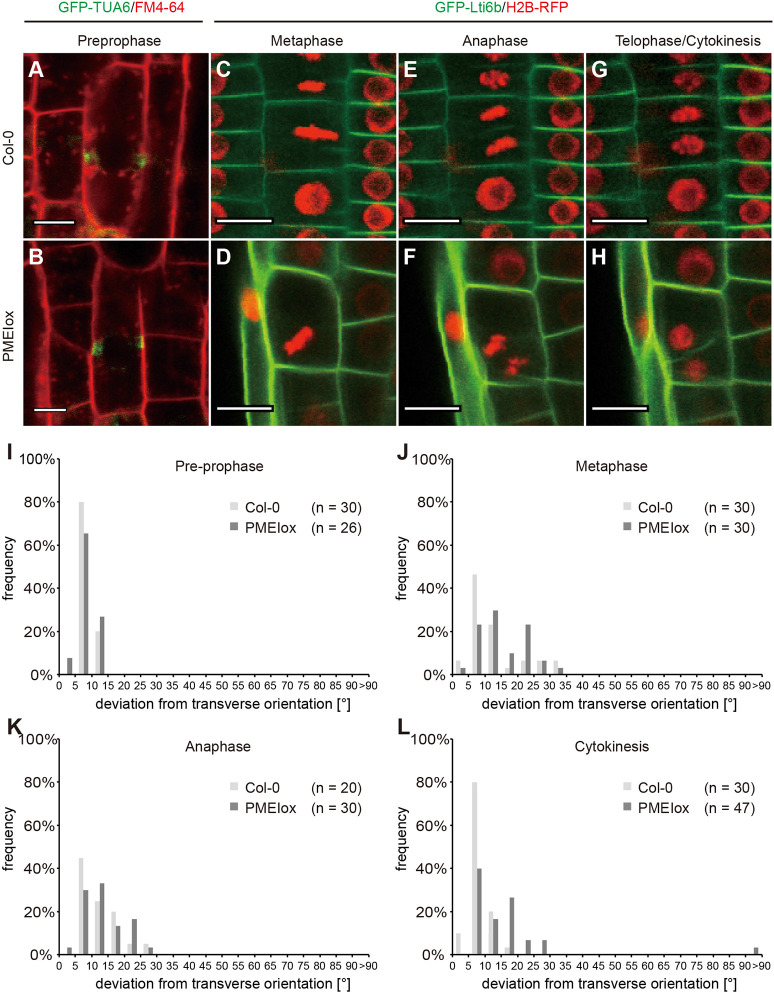
**Cell wall perturbation by PMEIox leads to cell division defects after specification of the cortical division zone.** (A,B) Orientation of pre-prophase bands labelled by GFP-TUA6 is transverse in both Col-0 (A) and PMEIox (B); see I for quantification. (C-F) Metaphase plate orientation (C,D; see J for quantification) and sister chromatid orientation during anaphase (E,F; see K for quantification) deviates from a 90°C angle in both the Col-0 wild type (C,E) and PMEIox (D,F). (G,H) Orientation of the cell plate in the Col-0 wild type stabilised at around 90°C relative to the cell long axis (G), whereas PMEIox cell plates showed a wide range of orientations (H) (see L for quantification). (I-L) Quantification of division plane orientation during mitosis and cytokinesis. Bars in histograms denote fraction of cells in bins bordered by angles indicated on the *x*-axis. *n*=30-50. Scale bars: 20 µm (A,C,E,G); 10 µm (B,D,F,H).

To test whether the aberrant cell wall placement in PMEIox was associated with defects in cell division plane orientation, we quantified the orientation of this plane at the different mitotic stages (Fig. 7). The PPB transiently indicates positioning of the CDZ (Rasmussen et al., 2013). Hence, to determine whether cell wall perturbations affect this positioning, we used a fluorescently labelled tubulin (GFP-TUA6) that allows visualisation of the PPB (Fig. 7A,B). Quantitative analysis of PPB orientation in wild-type cortex and epidermis cells revealed only minimal deviations from a position perpendicular to the cell long axis (Fig. 7I). PMEIox displayed a very similar distribution, indicating that division plane orientation defects are independent of PPB positioning (Fig. 7I). As the PPB disappears in pro-metaphase, we used a transgenic line expressing an RFP-fused Histone 2B to label chromatin and a GFP-fused plasma membrane protein Lti6b (Maizel et al., 2011) to visualise cell outlines and the forming cell plate. We quantified the orientation of the metaphase plate, the midline between sister chromatids, and the cell plate in metaphase, anaphase and telophase. Interestingly, the orientation of both the Col-0 wild-type and PMEIox metaphase plates deviated from a 90° angle relative to the cell long axis (Fig. 7C,D,J). A similar observation was made during anaphase (Fig. 7E,F,K). However, the forming cell plate during telophase was largely aligned with the expected division plane in Col-0, i.e. perpendicular to the cell long axis, and deviation angles appeared very similar to what was previously observed with the PPB (Fig. 7G,L). In contrast, telophase cell-plate orientation in PMEIox showed a distribution similar to what was observed in meta- and anaphase, consistent with defective re-alignment to the ∼90° angle observed in Col-0 (Fig. 7H,L). In summary, the PPB orientations in wild type and PMEIox appeared to be similar to each other, whereas both wild-type and PMEIox virtual division planes defined by the H2B marker deviated from transverse orientation during metaphase and anaphase. During telophase, wild-type cell plates largely aligned with the expected position perpendicular to the cell long axis, whereas a substantial fraction of PMEIox cell plates did not.

**Fig. 8.**
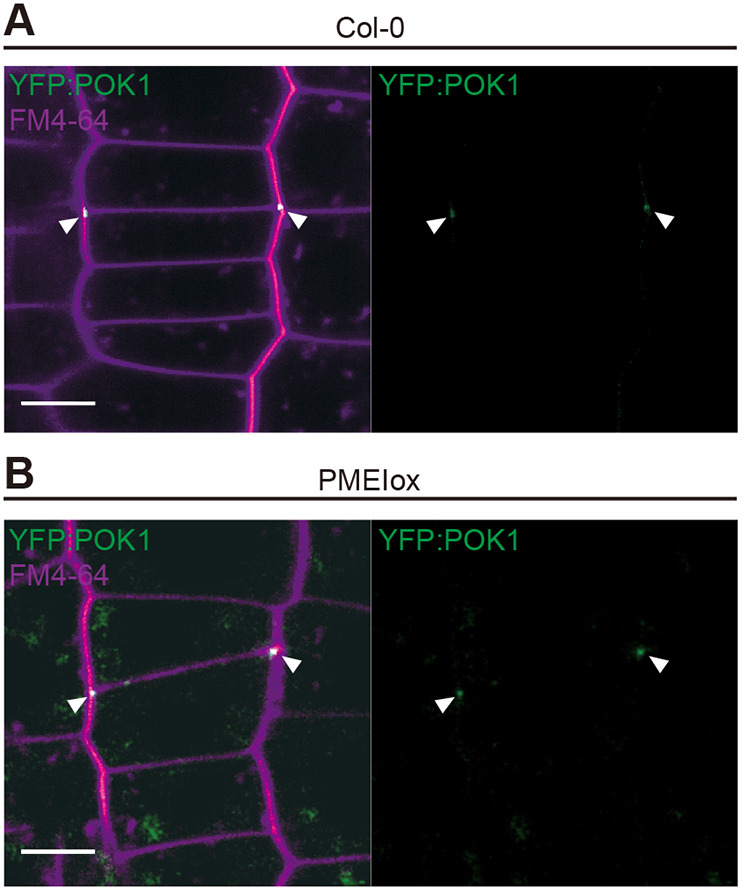
**Cell plate fusion in PMEIox coincides with POK1 localisation at aberrant positions.** (A,B) Cell plate fusion site position and YFP-POK1 localisation at the end of cytokinesis in Col-0 (A) and PMEIox (B). Note that the cell plate fusion at POK1-positive sites in the parental walls deviates from the normal position in PMEIox. White arrowheads indicate YPF-POK1-marked cell plate fusion sites. Membranes are labelled with FM4-64. Scale bars: 20 μm.

These observations are consistent with two hypotheses that could explain the aberrant cell wall angles in PMEIox. First, PMEIox cell plates might fail to find the CDZ initially marked by the PPB and occupied by POK1 and other components (Livanos and Müller, 2019). Second, the position of the cortical division zone might be mobile relative to the cell walls in PMEIox, although guidance is unaffected. To differentiate between the two possibilities, we introduced a fluorescently tagged version of POK1, YFP-POK1 (Lipka et al., 2014), into PMEIox and determined whether the cell plate fusion site coincided with the location of POK1 (Lipka et al., 2014; Müller et al., 2006). Cell plate fusion sites were marked by YFP-POK1, even when the cell plate fused at an angle deviating from 90° relative to the parental walls, suggesting that phragmoplast guidance towards CDZ components is unaffected in PMEIox (Fig. 8). As we did not observe aberrant PPB positioning, our results suggest that YFP-POK1, and thus potentially CDZ localisation, might deviate from the expected position during cytokinesis. Together, aberrant cell plate fusion in PMEIox coincides with POK1 localisation at positions that deviate from the cell midline. Our results show that optimal BR signalling strength is required to maintain the orientation of newly placed cell walls.

## DISCUSSION

Here, we report that cell wall integrity and BR signalling are involved in the control of cell wall orientation in the *Arabidopsis* root. Both pectin-triggered cell wall signalling, as in PMEIox plants, and impaired BR signalling resulted in aberrant cross walls in the *Arabidopsis* root meristem.

In PMEIox plants, pectin modification triggers an RLP44-mediated activation of BR signalling which, in turn, prevents loss of cell wall integrity, but results in a wide variety of growth-related phenotypes (Holzwart et al., 2018; Wolf et al., 2012, 2014; this study). Aberrant cell wall orientation due to an altered cell wall in PMEI5-expressing plants appears to occur downstream of BR signalling, as mutations in *BRI1* (*cnu1*, *bri1^cnu4^*) or *RLP44* (*cnu2*) mutants, largely suppressed the oblique cell wall phenotype of PMEIox. Analysis of PMEIox cell divisions at the subcellular level revealed that the virtual cell division plane between meta- and telophase, marked by the midline between chromatin structures (metaphase plate and segregating sister chromatids), showed deviations from the 90° angle in both wild-type and PMEIox cells. Wild-type cell plates later displayed the expected transversal orientation, whereas PMEIox cell plates frequently did not. Our use of metaphase plate and sister chromatids as a read-out for the ‘virtual’ cell division plane can be questioned as, for example, mitotic features such as spindle orientation do not always correlate with division plane orientation (Cleary and Smith, 1998; Galatis et al., 1984; Marcus et al., 2003; Oud and Nanninga, 1992; Rasmussen et al., 2011, 2013). However, that wild-type cell division plane orientations show considerable variation and noise, but are later harmonised by interaction with CDZ components, is in agreement with the phenotype of CDZ mutants such as *tan1*, *pok1 pok2* and *phgap1 phgap2* (Lipka et al., 2014; Müller et al., 2006; Smith et al., 1996; Stöckle et al., 2016; Walker et al., 2007). In these mutants, oblique cell walls are presumably the result of a lack of phragmoplast guidance. Our analysis suggests that PMEIox and Col-0 behave similarly with respect to all aspects analysed up until the very last steps of cell division, during which a substantial fraction of PMEIox cell plates fail to show orientation perpendicular to the cell long axis. Notably, it is conceivable that oblique cell walls in PMEIox occur through a mechanism different from that in the aforementioned CDZ mutants, as the CDZ in some PMEIox cells appears to have shifted from the expected position, based on YFP-POK1 localisation. This raises the issue of how the relative position of the CDZ is maintained in wild-type cells and whether this could involve interactions with the cell wall as previously suggested (Smertenko et al., 2017). Supporting an involvement of the cell wall in CDZ maintenance is the observation that four-way junctions comprising cross walls of adjacent cells fusing at a similar position of their shared longitudinal wall are actively avoided: PPBs are placed with an offset from the predicted division plane if a cross wall or PPB from an adjacent cell is found at this position and it is conceivable that the necessary signal to trigger this response involves the cell wall (Flanders et al., 1990; Martinez et al., 2018). In addition, cell wall attachment of the CDZ is a plausible way to maintain its relative position in the plasma membrane, in line with the observation that the cell wall is required for the maintenance of PIN polarity (Feraru et al., 2011) and acts to restrict the mobility of plasma membrane proteins in general (Martiniere et al., 2012; McKenna et al., 2019).

Whether there is a direct relationship between cell shape, in part controlled by cell division orientation, and organ shape is a long-standing question (Beemster et al., 2003; Hong et al., 2018; Kaplan and Hagemann, 1991; Martinez et al., 2017). Both continuous pectin-triggered cell wall signalling activation and exogenous application of BRs result in root growth alterations such as waviness and a reduction in root length (Lanza et al., 2012; Wolf et al., 2012; this study). We observed that cell type-specific induction of cell wall signalling separated root growth, its directionality and cell wall orientation defects based on which cell type expressed PMEI5. Furthermore, pectin-triggered cell wall signalling and reduced BR signalling in *bri1* mutants led to similar cell wall orientation defects in the meristem, but only PMEIox and plants treated with high BR concentrations displayed root waviness. In addition, plants expressing *PMEI5* in cortex cells showed pronounced cell division orientation defects without influencing organ morphology, growth, meristem size or meristematic cell number, whereas epidermal expression of BRI1 in *bri1*-triple mutants rescued organ level growth but enhanced cell division orientation defects, likely because of high BR signalling. Although cell wall orientation defects in BR loss-of-function mutants and in plants with enhanced BR signalling might occur through different mechanisms, these results demonstrate that aberrant placement of new walls is compatible with normal organ growth.

Reminiscent of previous findings demonstrating non-cell autonomous effects of BR signalling, cortical *PMEI5* expression led to oblique cell divisions in both epidermal and cortex cells. We cannot determine whether the non-cell autonomous effect is directly linked to PMEI5 or to downstream signalling components. However, it is noteworthy that expression of *PMEI5* in two spatially separated cell types, hair cells in the epidermis and the XPP cells, affects whole organ level responses (root waving). Future work needs to address how these organ-level responses are connected to cellular effects of BR signalling and how the cell wall is connected to cell division orientation maintenance, taking into account potential mechanical feedbacks and the contribution of cell geometry and developmental signalling (Besson and Dumais, 2011, 2014; Chakrabortty et al., 2018; Lintilhac and Vesecky, 1984; Louveaux et al., 2016; Martinez et al., 2018; Moukhtar et al., 2019; Yoshida et al., 2014).

The defects in cell wall orientation described here for BR receptor mutants appear to be random and it is unclear whether these cell division defects are related to previously described disturbed cell files and altered tissue organisation in rice *bri1* mutants (Nakamura et al., 2006). The use of a tissue-specific approach to perturb the BR signalling was an instrumental tool to disentangle the pleiotropic effects of the BR pathway. Hence, despite a ubiquitous expression of BRI1, it enabled the discovery of tissue-specific effects on shoot growth, root meristem size, final cell size, root length and gene expression that were otherwise masked by alternative overexpression or loss-of-function studies (Singh and Savaldi-Goldstein, 2015). Here, we show that aberrant cell wall orientation can be largely rescued in both *bri1* and *bri1*-triple mutant backgrounds while other growth parameters are poorly rescued, and vice versa. For example, *pGL2-BRI1* in *bri1*-triple has long root meristems and almost wild type-like root length and shows an increased number of anticlinal divisions (Hacham et al., 2011; Vragović et al., 2015) but harbours severe wall orientation defects. This could be the result of high local BR signalling intensity (Fridman et al., 2014; Vragović et al., 2015), as in pML1>GR>PMEI5. However, *pSHR-BRI1* has a shorter root and wide meristem (Vragović et al., 2015; Kang et al., 2017) that largely rescued these wall orientation defects. A tissue-specific approach recently also assisted in separating BRI1 control of phloem differentiation from that of growth (Graeff et al., 2020). As BRI1 is not expressed in the phloem in the lines analysed here, restoration of BR signalling in diverse cell types is sufficient to control root length and orientation of transversal walls.

Taken together, our results demonstrate that cell wall integrity and optimal BR signalling levels are required for a correct cell wall placement. This BR signalling-mediated control of cell wall orientation occurs both cell autonomously and non-cell autonomously and is uncoupled from organ-level growth control.

## MATERIALS AND METHODS

### Plant material

All genotypes used in this study were in the Col-0 background and are listed in Table S1. For PMEox-related experiments, seeds were sterilised with 1.3% (v/v) sodium hypochlorite (NaOCl) diluted in 70% ethanol for 3 min, then washed twice with 100% ethanol and dried in laminar flow hood. Seeds were sowed out on plate with growth medium containing half-strength (1⁄2) Murashige & Skroog (MS) medium (Duchefa), 1% D-sucrose (Car Roth) and 0.9% phytoagar (Duchefa) with pH adjusted to 5.8 with KOH. After 2 days stratification at 4°C in darkness, plates were placed vertically in long-day conditions (16 h light/8 h dark cycles) with equal light conditions (∼100 μE m^-2^s^-1^) for 5 days. All analyses have been carried out on 5-day-old seedlings. For DEX induction on plate, DEX (Sigma-Aldrich, D4902) was added to the growth medium, and an equal volume of dimethyl sulfoxide (DMSO) was added to the control plate. For DEX induction on soil, 30 μM DEX was used to spray the aerial part of the plant and to water every other day starting from 3 days after transfer of seedling on soil. All plants were grown on soil under long-day conditions (16 h light/8 h dark cycles) at 23°C with 65% humidity. For BRI1-related experiments (i.e. Fig. 5), seeds were sterilised and grown as described in Fridman et al. (2014).

### Microscopy

Microscopic analyses were carried out using Zeiss LSM 510 Meta, a Zeiss LSM710, and Leica TCS SP5 laser scanning confocal microscopes. For mTurquoise2, an excitation wavelength of 458 nm was used and emission was collected between 460 and 520 nm. GFP was excited with a 488 nm laser line and fluorescence was collected between 490 and 530 nm. mVenus was excited with a 514 nm laser line and fluorescence was collected between 520 and 560 nm. For propidium iodide (PI) fluorescence, an excitation wavelength of 488 nm was used, whereas emission was collected between 600 and 670 nm. RFP and FM4-64 fluorescence was excited at 561 nm and emission was collected between 560 and 620 nm (RFP) or between 675 and 790 nm (FM4-64). SCRI Renaissance 2200 was excited with the 405 nm laser and fluorescence was collected between 425 and 475 nm. Images were analysed with ImageJ/Fiji (https://imagej.net/software/fiji/).

### Plasmid construction

All constructs were produced using GreenGate cloning (Lampropoulos et al., 2013) with modules and primers listed in Table S2. PCR products were generated using Q5^®^ High-Fidelity DNA Polymerase (NEB) and column-purified by using GeneJET PCR Purification Kit (Thermo Fisher Scientific), followed by restriction digest with Eco31I FD restriction enzyme (Thermo Fisher Scientific) at 37°C for 15 min. Products were column-purified as described above. Empty entry vectors (pGGA000, pGGC000; Lampropoulos et al., 2013) were digested and purified separately. Digested and purified insert and vector were ligated using Instant Sticky-end Ligase Master Mix (New England Biolabs) following the manufacturer's instructions. The ligation products were then used to transform chemically competent *Escherichia coli* strain DH5α or XL1-blue and cultivated in LB medium supplied with Ampicillin. Plasmid sequences were verified by Sanger sequencing. Confirmed entry modules are ligated into intermediate vectors by using a GreenGate reaction (Lampropoulos et al., 2013). The generation of expression plasmids involved the creation of two intermediate constructs, one in pGGM000 carrying the GR-LhG4 expression cassette, and one in pGGN000 carrying the PMEI5 coding sequence under control of the pOp6 promoter. The assembly of two expression cassettes each carried by one intermediate vector was achieved by running the same GreenGate reaction and replacing the entry module and empty intermediate vector by an intermediate module and an empty pGGZ001 destination vector, respectively. Final plasmids were verified by colony PCR and restriction digest, and then used to transform *Agrobacterium tumefaciens* strain ASE (pSOUP+) carrying resistance to chloramphenicol, kanamycin and tetracycline. All constructs were transformed by the floral dip method as previously described (Clough and Bent, 1998; Zhang et al., 2006).

### Root staining and analysis

Staining of cell outlines with PI (Sigma-Aldrich, P4170) using a modified pseudo-PI staining method has been performed as described in Truernit et al. (2008), with modifications. Seedlings at 5 DAG were fixed in solution containing 50% methanol and 10% acetic acid for 3 days at 4°C. Samples were then washed twice with H2O and incubated in 1% periodic acid (Sigma-Aldrich, P0430) at room temperature for 40 min. Samples were washed twice with H2O and then stained with 100 μg/ml PI freshly diluted in Schiff's reagent (100 mM sodium metabisulphite, 75 mM HCl). Stained samples were transferred onto microscope slides covered by chloral hydrate solution (4 g chloral hydrate, 1 ml glycerol, 2 ml H2O) and incubated overnight at room temperature in a closed environment. Excess of chloral hydrate solution was removed and several drops of Hoyer's solution (3 g gum arabic, 20 g chloral hydrate, 2 g glycerol, 5 ml H2O) was added to the samples, which were covered gently by cover slips and kept at room temperature for 3 days before imaging. Alternatively (Fig. S1), roots were fixed in paraformaldehyde and stained with SCRI Renaissance 2200 (Renaissance Chemicals) as previously described (Musielak et al., 2015) and cleared in ClearSee (Kurihara et al., 2015). Samples were mounted in ClearSee using imaging chambers.

Orientation of mitotic structures, RAM length and meristematic cell number were measured using ImageJ. The meristem was defined as the region between the quiescent centre and the first cortex cells with twice the length of the previous cell. Directional growth of the roots was analysed by determining the vertical growth index, i.e. the distance of base of the root to the tip divided by root length. Quantification of aberrant cell wall orientation per root was performed by counting the number of cross walls with >15° deviance from transverse orientation in median confocal sections. If not stated otherwise, only cortex cell files were considered. In diagrams, stacked bars denoting more than three aberrant cell walls per root are assigned the same colour for clarity, but statistics were performed with the actual numerical and un-binned values.

### QRT-PCR analysis

Seedling roots were harvested at 5 DAG and directly frozen in liquid nitrogen. After tissue homogenisation, RNA extraction was carried out using the GeneMATRIX Universal RNA Purification Kit (EURx, 3598), following the manufacturer's instructions. cDNA synthesis and QRT-PCR analysis was performed as previously described (Holzwart et al., 2020). PMEI5 cDNA was amplified with primers 5′-ACGTGCTTTTAATTGCGATAACG-3′ and 5′-GAAGTCCAAGTTCCCAAGCTG-3. All experiments have been repeated at least twice, with identical results.

## Supplementary Material

Supplementary information

Reviewer comments
