# Supplementary material for: Optimal BR signalling is required for adequate cell wall orientation in the Arabidopsis root meristem
Source: Development. 2021 Nov 10;148(21):dev199504. doi: 10.1242/dev.199504 (PMC8627601; doi:10.1242/dev.199504)
Supplement: Supplementary information [file develop-148-199504-s1.pdf]

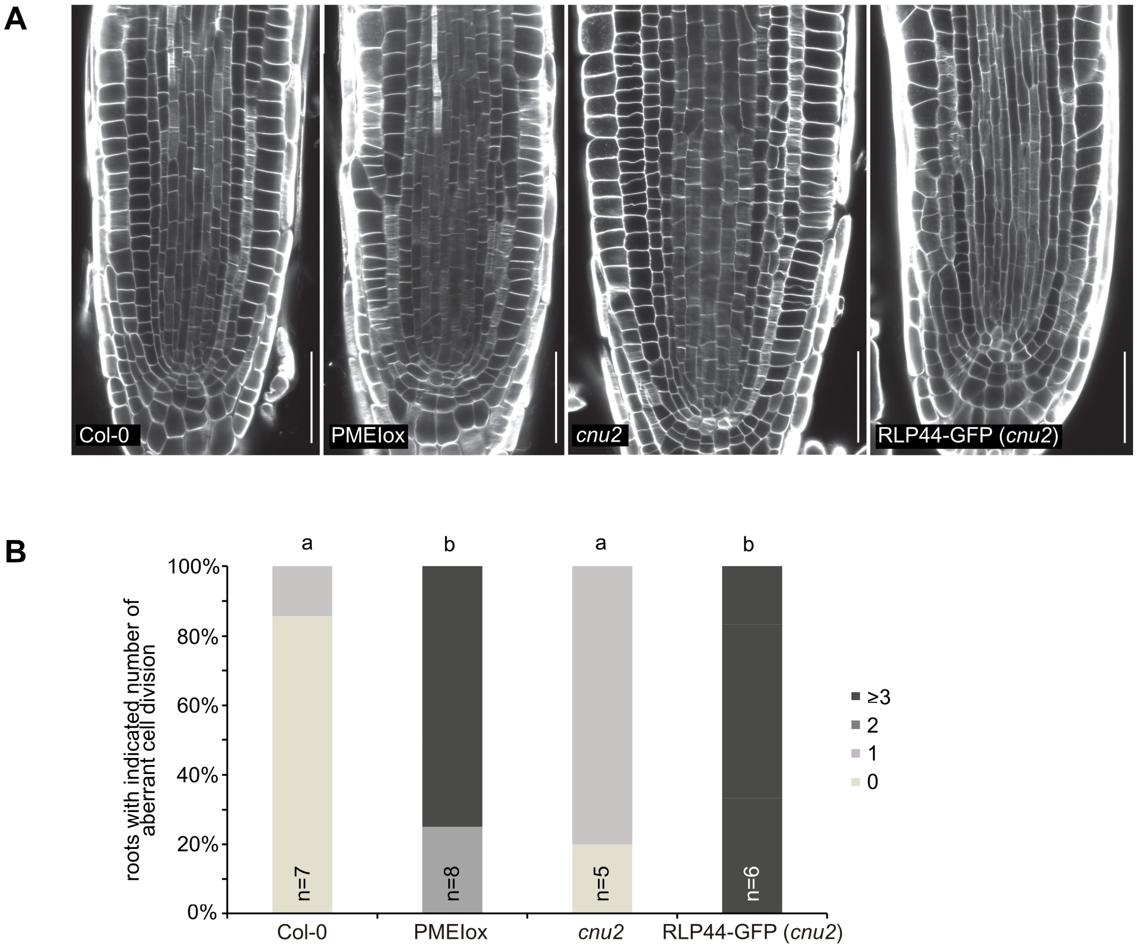

Fig. S1. Expression of RLP44-GFP in *cnu2* mutants restores aberrant cell division plane orientation. (A) Median sections through root tips of Col-0, PMEIox, the PMEIox suppressor mutant *cnu2,* and a complemented line expressing RLP44-GFP in the *cnu2* background. Scale bars = 50 µm. (B) Quantification of the fraction of roots with the indicated number of oblique transversal walls in cortex cells from confocal sections. Letters indicate statistically significant differences according to Dunn's test after Kruskal-Wallis analysis.

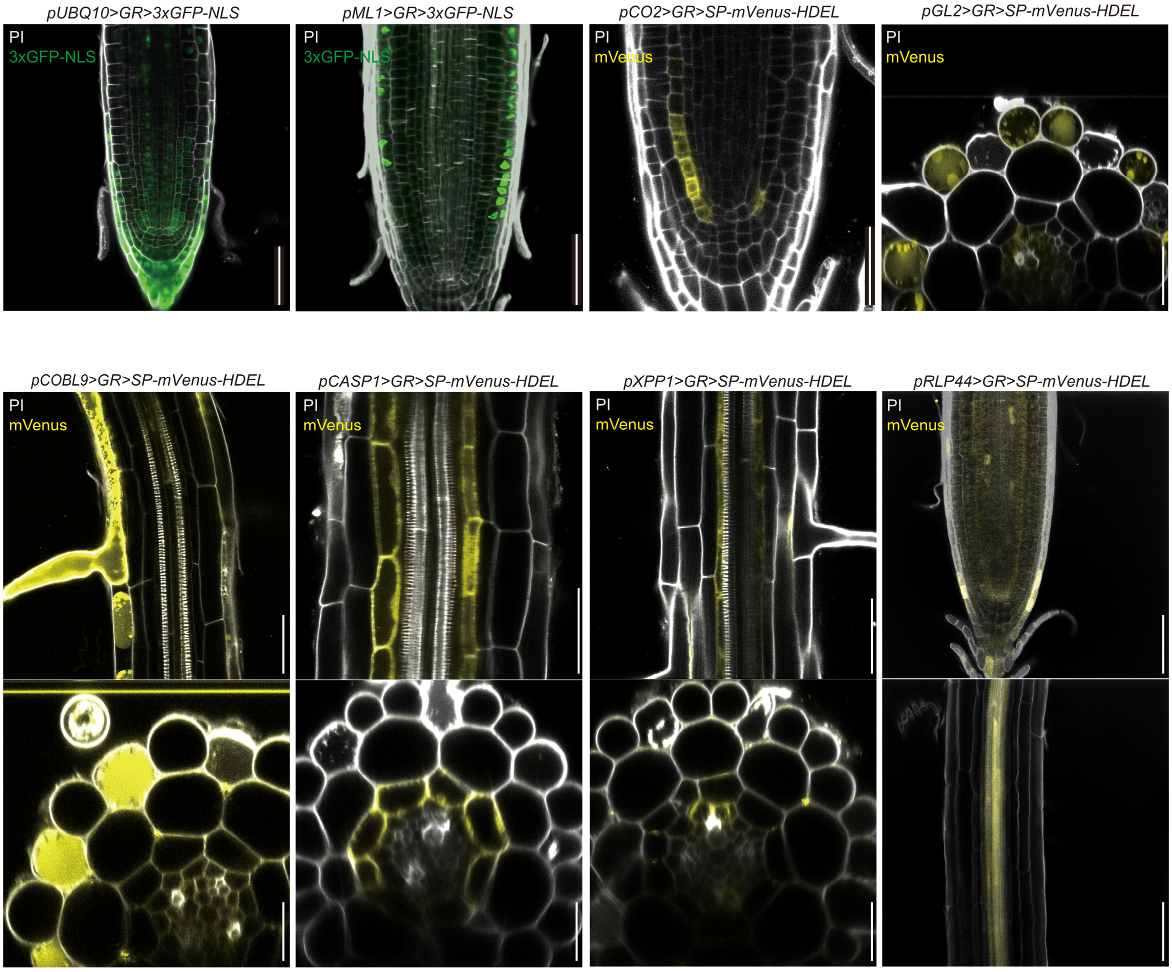

Fig. S2. Fluorescent reporters under control of the pOp promoter demonstrating tissue-specific expression in GR-LhG4 trans-activation lines. Fluorescence derived from the indicated reporter (pOp6:3xGFP-NLS or pOp6:SP-mVenus-HDEL) in lines expressing the GR-LhG4 transcription factor in the epidermis (under control of the *pML1* promoter), in meristematic cortex cells *(pCO2*), in trichoblasts (*pCOBL9),* atrichoblasts (*pGL2*), in differentiating and mature endodermis cells (*pCASP1)*, in xylem pole pericycle cells (*pXPP)*, and in most RAM tissues with highest expression in the vascular tissue under control of the *pRLP44* promoter. Cells walls are counter-stained with PI. Bars = 50 µm in longitudinal sections and = 20 µm in cross sections. Cross sections are from the differentiation zone of the root before formation of the casparian strip.

Development • Supplementary information

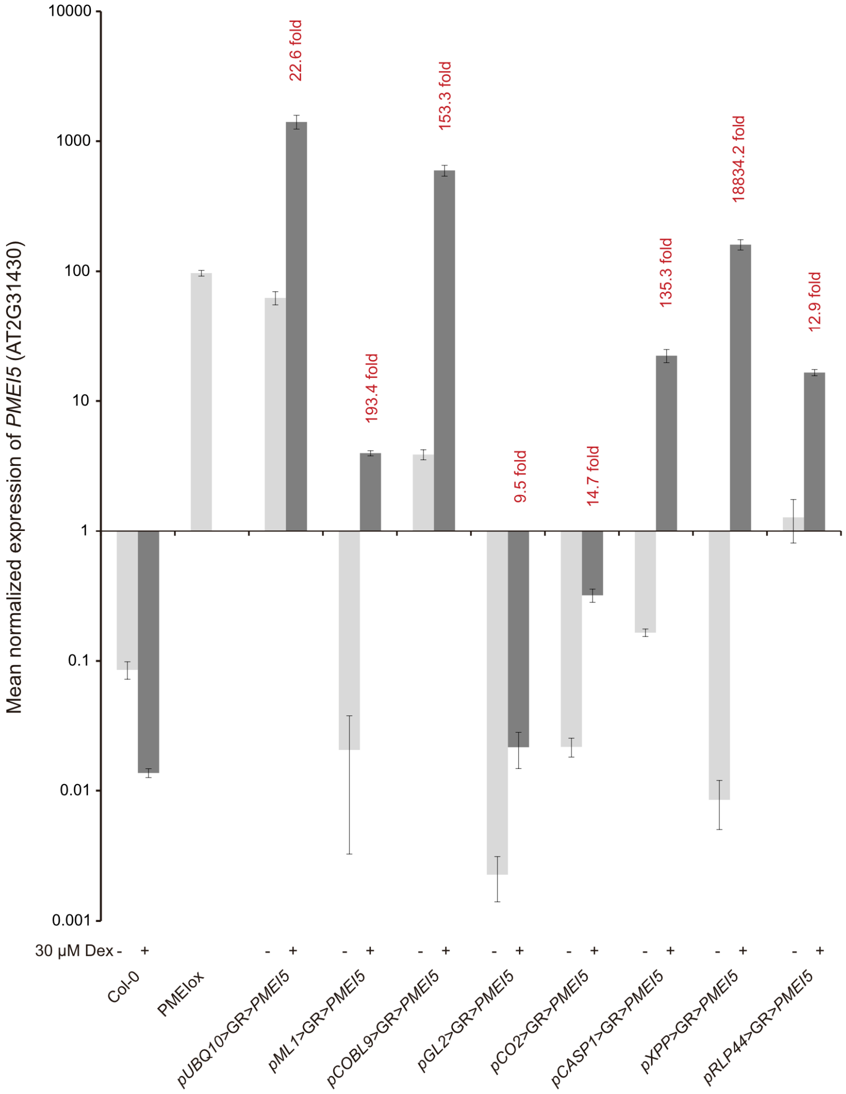

Fig. S3. Induction of PMEI5 expression after DEX treatment in trans-activation lines. Values denote mean normalized expression with at5g46630 as reference gene. Fold changes compared to the uninduced control are indicated, n = 3, bars indicate mean ± s.e.m.

Development • Supplementary information

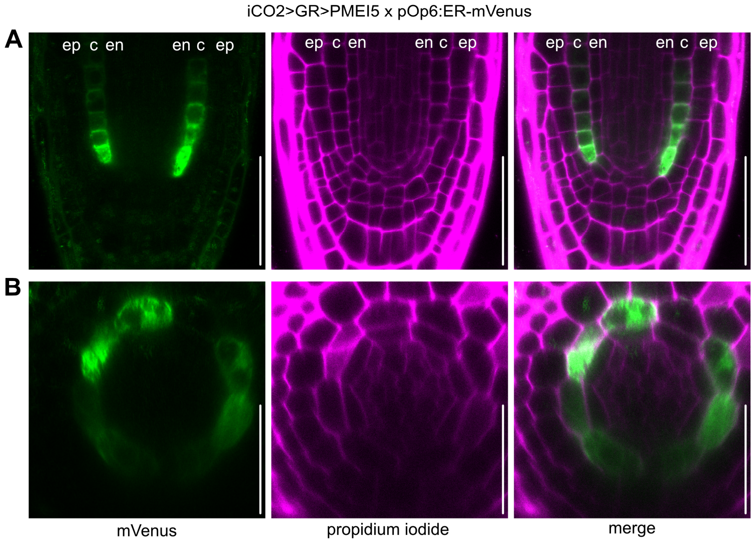

Fig. S4. Simultaneous trans-activation of PMEI5 and a pOp6-driven, ER-localized reporter demonstrates cortex-specific expression mediated by the pCO2> driver line. Bars = 50 µm, ep = epidermis, c = cortex, en = endodermis.

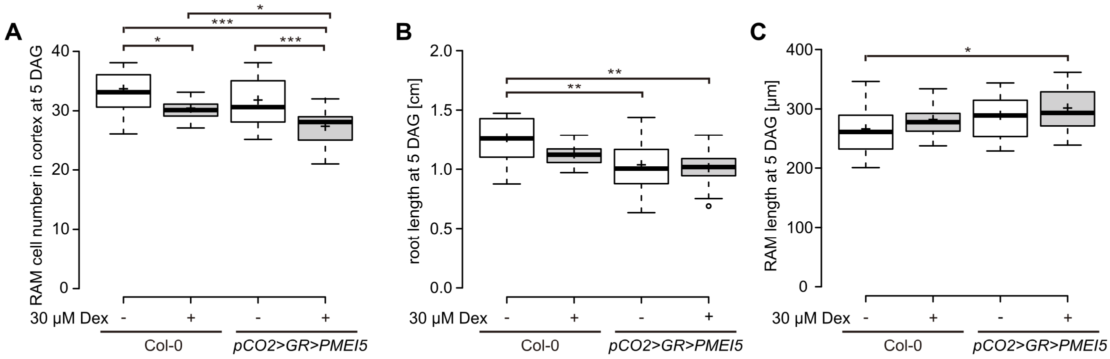

Fig. S5. Expression of *PMEI5* in meristematic cortex cells (pCO2>GR>PMEI5) had only mild effect on primary root growth and RAM morphology. Primary root length (A), RAM cell number (B), and RAM length (C) five days after germination in Col-0 and pCO2>GR>PMEI5 roots in the absence and presence of the inducer DEX. n = 40 - 50. Asterisks indicate statistically significant difference revealed by Dunn's test following a Mann-Whitney U test, p <0.05 (*), p < 0.01 (**) and p < 0.001 (***).

Development • Supplementary information

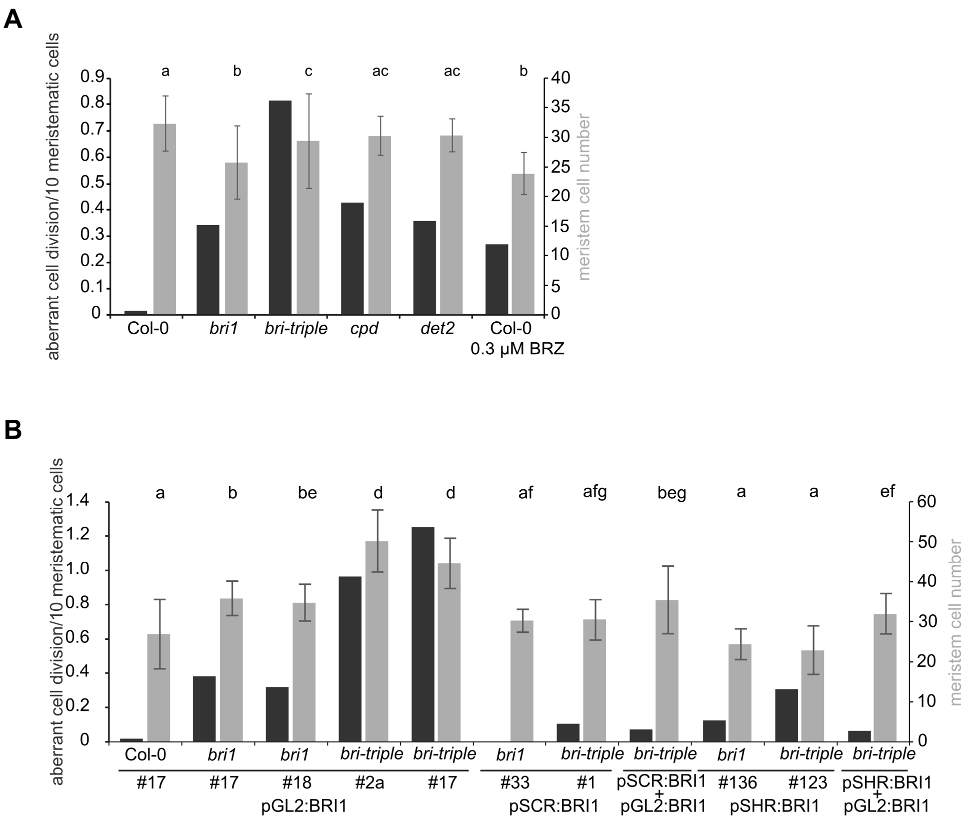

Fig. S6. Aberrant cell divisions in meristems of BR mutants and plants grown under BR-limiting conditions, corresponding to Figure 5. Dark bars show number of aberrant cell divisions in the meristems of the indicated genotypes per 10 meristematic cortex cells. Light grey bars (secondary y-axis) indicate average meristematic cell number ±S.D. Letters indicate statistically significant difference among genotypes with respect to meristematic cell number according to Dunn's test following Kruskal Wallis test. Analysis was performed on samples used for Figure 5.

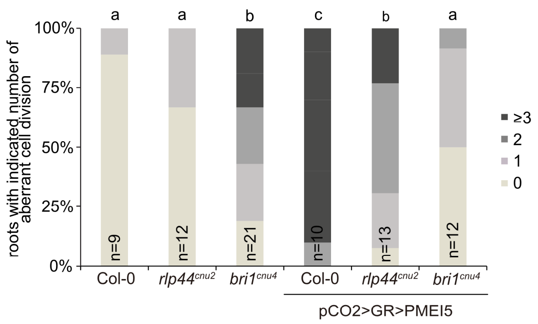

Fig. S7. Aberrant cell wall orientation in pCO2>GR>PMEI5 are RLP44- and BRI1-dependent. Quantification of the fraction of roots with the indicated number of oblique transversal walls in cortex cells from confocal sections. Letters indicate statistically significant differences according to Dunn's test after Kruskal-Wallis analysis.

Development • Supplementary information

Table S1. Mutants and transgenic lines used in this study

Mutant/transgenic line	Reference	Accession
PMEIox	Wolf et al., 2012	
cnu1	Wolf et al., 2012	
cnu2	Wolf et al., 2014	
pUBQ10>GR>PMEI5	this study	
pML1>GR>PMEI5	this study	
pCOBL9>GR>PMEI5	this study	
pGL2>GR>PMEI5	this study	
pCO2>GR>PMEI5	this study	
pCASP1>GR>PMEI5	this study	
pXPP>GR>PMEI5	this study	
pRLP44>GR>PMEI5	this study	
pOp6:ER-Venus	Schürholz et al., 2018	
bri1-116	Li and Chory 1997	
bri triple	(Vragovic et al., 2015)	*bri1-116*; SALK_005982 (brl1); SALK_006024 (brl3)
pGL2:BRI1-GFP	Fridman et al., 2014	
pGL2:BRI1-GFP (bri1-116)	Hacham et al., 2011	
pGL2:BRI1-GFP (bri-triple)	Vragovic et al., 2015	
pSCR:BRI1-GFP (bri1-116)	Hacham et al., 2011	
pSCR:BRI1-GFP (bri-triple)	Vragovic et al., 2015	
pSHR:BRI1-GFP (bri1-116)	Hacham et al., 2011	
pSHR:BRI1-GFP (bri-triple)	Vragovic et al., 2015	
cpd	Szekeres et al., 1996	
det2	Chory et al., 1991	
GFP-TUA6	Ueda et al., 1999	
GFP-LTI6b/H2B-RFP	*Maizel et al., 2011*	
35S:RLP44-GFP (cnu2)	Wolf et al., 2014	

Table S2. Overview of constructs generated with GreenGate cloning (Lampropoulos et al., 2013) and the primers used to generate modules, where appropriate.

**pSW507**	pUBQ10 intermediate	
pSW336	pUBQ10 (pGGA0006)	Lampropoulos et al., 2013
pSW182	B-Dummy (pGGA022)	Lampropoulos et al., 2013
pSW181	GR-LhG4	Schürholz et al., 2018
pSW184	D-dummy (pGGD002)	Lampropoulos et al., 2013
pSW185	Rbcs terminator (pGGE001)	Lampropoulos et al., 2013
pSW188	F-H adapter	Lampropoulos et al., 2013
pGGM000	Intermediate vector	Lampropoulos et al., 2013
**pSW304**	pML1 intermediate	
pSW179	pML1 (pGGA0022)	Schürholz et al., 2018
pSW182	B-Dummy (pGGA022)	Lampropoulos et al., 2013
pSW181	GR-LhG4	Schürholz et al., 2018
pSW184	D-dummy (pGGD002)	Lampropoulos et al., 2013
pSW185	Rbcs terminator (pGGE001)	Lampropoulos et al., 2013
pSW188	F-H adapter	Lampropoulos et al., 2013
pGGM000	Intermediate vector	Lampropoulos et al., 2013
**pSW306**	pCOBL9 intermediate	
pSW222	pCOBL9	AACAGGTCTCAACCTTATGTACTCTTTTAGTGGTTTACAC<br>AACAGGTCTCATGTTTGTGTCTTTCTCCAGAGAAAG
pSW182	B-Dummy (pGGA022)	Lampropoulos et al., 2013
pSW181	GR-LhG4	Schürholz et al., 2018
pSW184	D-dummy (pGGD002)	Lampropoulos et al., 2013
pSW185	Rbcs terminator (pGGE001)	Lampropoulos et al., 2013
pSW188	F-H adapter	Lampropoulos et al., 2013
pGGM000	Intermediate vector	Lampropoulos et al., 2013
**pSW307**	pGL2 intermediate	
pSW223	pGL2	AACAGGTCTCAACCTCGTATTATACGGACGGTTTAAGC<br>AACAGGTCTCATGTTTTTTCTTCTTAATATTCGATTTTTAATA
pSW182	B-Dummy (pGGA022)	Lampropoulos et al., 2013
pSW181	GR-LhG4	Schürholz et al., 2018
pSW184	D-dummy (pGGD002)	Lampropoulos et al., 2013
pSW185	Rbcs terminator (pGGE001)	Lampropoulos et al., 2013
pSW188	F-H adapter	Lampropoulos et al., 2013

Development • Supplementary information

pGGM000	Intermediate vector	Lampropoulos et al., 2013
**pSW305**	pCO2 intermediate	
pSW299	pCO2	AACAGGTCTCAACCTGATCAGAGTATTGGGCCTTTTGG<br>AACAGGTCTCATGTTTATCGTTATTAACTAGGGTTCTTG
pSW182	B-Dummy (pGGA022)	Lampropoulos et al., 2013
pSW181	GR-LhG4	Schürholz et al., 2018
pSW184	D-dummy (pGGD002)	Lampropoulos et al., 2013
pSW185	Rbcs terminator (pGGE001)	Lampropoulos et al., 2013
pSW188	F-H adapter	Lampropoulos et al., 2013
pGGM000	Intermediate vector	Lampropoulos et al., 2013
**pSW302**	pCASP1 intermediate	
pSW177	pCASP1	Schürholz et al., 2018
pSW182	B-Dummy (pGGA022)	Lampropoulos et al., 2013
pSW181	GR-LhG4	Schürholz et al., 2018
pSW184	D-dummy (pGGD002)	Lampropoulos et al., 2013
pSW185	Rbcs terminator (pGGE001)	Lampropoulos et al., 2013
pSW188	F-H adapter	Lampropoulos et al., 2013
pGGM000	Intermediate vector	Lampropoulos et al., 2013
**pSW303**	pXPP intermediate	
pSW178	pXPP	Schürholz et al., 2018
pSW182	B-Dummy (pGGA022)	Lampropoulos et al., 2013
pSW181	GR-LhG4	Schürholz et al., 2018
pSW184	D-dummy (pGGD002)	Lampropoulos et al., 2013
pSW185	Rbcs terminator (pGGE001)	Lampropoulos et al., 2013
pSW188	F-H adapter	Lampropoulos et al., 2013
pGGM000	Intermediate vector	Lampropoulos et al., 2013
**pSW308**	pRLP44 intermediate	
pSW299	pRLP44	AACAGGTCTCAACCTTTTGCGATATTTTTGGCTGTC<br>AACAGGTCTCATGTTTTTTAAATTTTAGAGAGGTTTC
pSW182	B-Dummy (pGGA022)	Lampropoulos et al., 2013
pSW181	GR-LhG4	Schürholz et al., 2018
pSW184	D-dummy (pGGD002)	Lampropoulos et al., 2013
pSW185	Rbcs terminator (pGGE001)	Lampropoulos et al., 2013
pSW188	F-H adapter	Lampropoulos et al., 2013
pGGM000	Intermediate vector	Lampropoulos et al., 2013
**pSW301**	PMEI5 intermediate	
pSW189	H-A adapter	Lampropoulos et al., 2013
pSW180	pOp6 (pGGA016)	Lampropoulos et al., 2013
pSW182	B-Dummy (pGGA022)	Lampropoulos et al., 2013

Development • Supplementary information

pSW190	PMEI5	AACAGGTCTCAGGCTCAATGGCCACAATGCTAATAAACCAC AACAGGTCTCACTGATTACTTATTTTCAACAAGCTTGTGACC
pSW184	D-dummy (pGGB003)	Lampropoulos et al., 2013
pSW186	UBQ10 terminator (pGGE009)	Lampropoulos et al., 2013
pSW187	KanR (pGGF007)	Lampropoulos et al., 2013
pGGN000	Intermediate vector	Lampropoulos et al., 2013

Click here to download Table S2

Development • Supplementary information
