# Supplementary material for: Optimal BR signalling is required for adequate cell wall orientation in the Arabidopsis root meristem
Source: Development. 2021 Nov 10;148(21):dev199504. doi: 10.1242/dev.199504 (PMC8627601; doi:10.1242/dev.199504)
Supplement: Reviewer comments [file develop-148-199504_review_history.pdf]

Development

Optimal BR signalling is required for adequate cell wall orientation in the Arabidopsis root meristem

Zhenni Li, Ayala Sela, Yulia Fridman, Lucía Garstka, Herman Höfte, Sigal Savaldi-Goldstein and Sebastian Wolf
DOI: 10.1242/dev.199504

Editor: Ykä Helariutta

Review timeline

Original submission:	5 February 2021
Editorial decision:	8 March 2021
First revision received:	16 June 2021
Editorial decision:	19 July 2021
Second revision received:	18 August 2021
Accepted:	4 October 2021

Original submission

First decision letter

MS ID#: DEVELOP/2021/199504

MS TITLE: Optimal BR signalling is required for adequate cell wall orientation in the Arabidopsis root meristem

AUTHORS: Zhenni Li, Ayala Sela, Yulia Fridman, Herman Hofte, Sigal Savaldi-Goldstein, and Sebastian Wolf

I have now received all the referees' reports on the above manuscript, and have reached a decision. The referees' comments are appended below, or you can access them online: please go to BenchPress and click on the 'Manuscripts with Decisions' queue in the Author Area.

As you will see, the referees express considerable interest in your work, but have some significant criticisms and recommend a substantial revision of your manuscript before we can consider publication. If you are able to revise the manuscript along the lines suggested, which may involve further experiments, I will be happy receive a revised version of the manuscript. Your revised paper will be re-reviewed by one or more of the original referees, and acceptance of your manuscript will depend on your addressing satisfactorily the reviewers' major concerns. Please also note that Development will normally permit only one round of major revision.

We are aware that you may be experiencing disruption to the normal running of your lab that make experimental revisions challenging. If it would be helpful, we encourage you to contact us to discuss your revision in greater detail. Please send us a point-by-point response indicating where you are able to address concerns raised (either experimentally or by changes to the text) and where you will not be able to do so within the normal timeframe of a revision. We will then provide further guidance. Please also note that we are happy to extend revision timeframes as necessary.

Please attend to all of the reviewers' comments and ensure that you clearly highlight all changes made in the revised manuscript. Please avoid using 'Tracked changes' in Word files as these are lost in PDF conversion. I should be grateful if you would also provide a point-by-point response detailing

© 2021. Published by The Company of Biologists under the terms of the Creative Commons Attribution License (https://creativecommons.org/licenses/by/4.0/).

how you have dealt with the points raised by the reviewers in the 'Response to Reviewers' box. If you do not agree with any of their criticisms or suggestions please explain clearly why this is so.

Reviewer 1

Advance summary and potential significance to field

In the manuscript by Li and co-workers, the authors connect BR signaling and cell wall orientation in the model species Arabidopsis.

Comments for the author

Besides the scientific remarks indicated below, the manuscript is kind of hard to read due to unnecessary long and complicated sentences. I would advise a serious rework to make the text more easy to read.

Additionally, figures are not of the highest quality (might be a pdf conversion problem) and there is a general sloppiness (not being precise) in the manuscript and figures. For example the text is full of non-scientific expressions such as “slightly”, “mild” etc. Results are either significant if proper statistical testing is done; or they are not. In one of the figures (Fig2E) dealing with root waving, there is what seems to be a bacterial contamination on the plate and the root closest to this shows a wave effect, while the other do not.

Obviously this makes any interpretation hard and gives the impression this experiment was only done once. It that respect, how many times were these experiments repeated?
Related to this, there is no indication how often the oblique divisions occur in different lines/conditions, or how many roots/experiments each image is representative of in Fig1-4. This should be clearly mentioned and should include a number of samples large enough to do proper statistical testing. This info must be added and the frequency of oblique divisions in different tissues, per root or per. e.g. 100 cells of the given type should also be included, to assess the strength of the phenotypes, similarly to what is done in Fig5. Now the oblique divisions in Fig1-4 are presented as a binary phenotype (oblique divisions yes/no), while for example the phenotype of constitutive PMEIOX (Fig1C) and the conditional one (3A) are very different in strength.

Could the authors additionally give an explanation for this?

Also related to this general remark, some statistics: t-test (Fig.S1A, S4) and ANOVA (5A) are reported. These are only valid for normally distributed data – has the normality of distribution been tested? If data is not normal, other types of tests must be applied (Wilcoxon, Kruskal-Wallis..). Can the authors please evaluate this for all their experiments and data? Also, the significance of categorical data (Fig.5B,C and similar quantifications from other experiments – see above) should also be tested by e.g. the CHI-squared test.

On line 316-318, to optimally support the statement made, time-lapse imaging of PMEIOx plants expressing markers of both POK1 and MTs would have to be performed (e.g. in an F1 cross of PMEI-OX with YFP-POK1;

RFP-MBD (Lipka et al., 2014). These lines are published and making the cross with analysis in F1 would be feasible. Currently, the statement is very bold based on the available data.
For the tissue specific complementation experiments, there is no control to validate these lines are functional.
E.g. Fig2F shows no effect by inducing PMEI5 in the endodermis, but there is no way of knowing this line is functional, so we could be looking at WT plants. Did the authors include a GFP tag in these lines to verify they are properly expressing the construct. Alternatively, did the authors validate the induction of the lines via Q-RT-PCR or an equivalent method? Given the importance given to these results, these lines would need to be validated before using.

Lines 294-299: formally the argument about alignment of cell division with the PPB cannot be made if the two are not analyzed in the same cells. Moreover, time-laps analysis with quantification

© 2021. Published by The Company of Biologists under the terms of the Creative Commons Attribution License (https://creativecommons.org/licenses/by/4.0/).

would also make a stronger point. The authors should consider making the cell biological data more convincing as suggested or alternatively reduce the strength of their claims and replace PPB position by CDZ or similar. Line 299 implies that cell plate positioning is defective in ALL PMEIOx cells, which is not true – please reword.

Smaller remarks that need to be addressed:
Fig.5C, bri1-116 pSHR::BRI1 – n=3 is not acceptable; increase the sample size or remove
Fig.5D pSHR-BRI: some roots show rescue and others do not. Can the authors explain this?
Line 51-52: is this precise?
Fig.1: what does the asterisk mean? Not in the legend. Also, no mentioning of WT grown on BL in the text (panel A). Moreover, there is no correlation between the root waving phenotypes in panel A and the cellular phenotypes in panels B-E. Next, line 143 (Legend Fig 1) – some strange typo. Also, preferably show 3-4 seedlings per genotype as they grew on the plate
Line 150: why only complementation of cnu2, but not cnu1, at this moment, even though the conclusion at the end of the paragraph covers both BRI and RLP44.
Line 180: yes it is waving, but in a way very different from the other lines
Line 185-186: This statement is likely correct in the context of your experiment, but the statement you make here is rather general. Please rephrase to make sure you are not generalizing your conclusions throughout the text.
Fig.3: DEX in B, mock in D are very bad images, as if chloral hydrate diffused into the water immersion. The quality of these images should be improved.
Lines 195-196: pCO2 is reported not to be entirely specific to cortex, so for claims of non-cell autonomous action its expression domain should be analyzed closely, or at least the possibility of leaky expression should be mentioned. Also on these lines, the phenotypes are described for specific cell files, although I also see defects in others. This all shows this phenotype needs to be much more closely evaluated and quantified to allow these statements to be made.
Figure4: Root meristems only reach their steady state growth rate around 5-6 days after germination, yet growth experiments were performed on very young plants: e.g. 2days. What is the impact of analyzing such young plants for growth defects? Also in Fig4 and others, the resolution/magnification of the figures is not good enough to evaluate the phenotypes described.
Line 211-212: What does “an oblique newly wall orientation defects” mean? Please revise this sentence. Same for Line 214-216: I do not understand these sentences.
Line 242: why spatiotemporal, where is the temporal effect?
Line 257: you can only talk about correlation if you do statistical testing. Do you mean perhaps uncoupled or unlinked?
Fig6: these observations are done in different cell files, while the authors have shown there can be a difference in response between them. Did the authors take this into account?
Figure S5 is difficult to read. it would be helpful to display the meristem size as bars in a different color and a scale on a secondary Y-axis. Significance of the data (oblique divisions per 10 cells) should be tested by CHI-
squared test as outlined in the major comments

Reviewer 2

Advance summary and potential significance to field

This manuscript by Li et al. reported the involvement of BR signaling in cell wall orientation in Arabidopsis roots. Cell division orientation is one of the key mechanisms that regulate tissue patterning during development. This is particularly important during plant organogenesis as all plant cells are encased in rigid cells. Previous studies from the same lab have shown that impaired physical properties of the cell wall promote BR signaling. They found that overexpression of pectin methylesterase inhibitor protein 5 (PMEI5) could activate BR signaling through RLP44, resulting in developmental phenotypes including organ fusion and root waving. In this paper, Li et al. made efforts to further connect the oblique cell division found in PMEI5 overexpression lines to BR signaling. This is a very interesting observation and most experiments were nicely designed and well performed. However, I think substantial revision is required to make it a publication at Development. My major concerns are as follows:

© 2021. Published by The Company of Biologists under the terms of the Creative Commons Attribution License (https://creativecommons.org/licenses/by/4.0/).

Comments for the author

Major concerns:

1. In figure 1, they showed altered cell wall orientation in PMEIox roots was largely restored in mutants with lesions in BRI1 (cnu1) and RLP44 (cnu2).
Since not all cells are affected, quantitative analysis of oblique cell wall with proper statistics is needed here to evaluate the extent of cell division phenotypes in different backgrounds. In addition, if the elevated BR signaling is the key translator of the cell wall defects in cell division, they should also be able to find oblique cell wall in brassinolide treated roots.

2. In the title and also many other places in the paper, the authors claimed that optimal BR signaling is important. However, I did not find any data showing the examination of BR signaling strength. In Figure 1, have they examined the effect of different concentrations of brassinolide? Can they find the optimal level that BR signaling promotes the cell division defect?

3. In figure 2, they inducibly mis-expressed PMEI5 in different cell types. How can they prove the correct expression of the gene? Why did they add a fluorescent tag to the PMEI5? Or if the disruption of the protein function is s concern, they should presented other evidence showing the successful mis-expression of PMEI5.

4. Among different lines (pUBQ10>GR>PMEI5, pML1>GR>PMEI5, pCOBL9>GR>PMEI5 and pXPP>GR>PMEI5), they only found pUBQ10>GR>PMEI5 roots had both root waving phenotypes and defective cell wall placement. However, BR level should be elevated in all four lines. Have they performed any quantification to dissect if the phenotypes were caused by distinct BR level in different lines, or by different spatial distribution?

5. Interestingly, when specifically expressed in cortex (pCO2>GR>PMEI5), misexpressed PMEI led to oblique cell walls without occurrence of root waving. However, the cell wall phenotype seemed to occur in both cortex and epidermis.
Could they explain this phenomenon? In addition, the phenotype appeared not as dramatic as in PMEIox lines. Is that because of the expression level?
Quantification of the cell wall phenotype should be provided.

6. I found the variation of oblique phenotypes in the images they provided. In figure 1c, PMEIox roots had very strong cell wall defects, while pUBQ10>GR>PMEI5 root in figure 3A seemed to have fairly mild phenotype. Again I think the authors should make it clear that how they evaluate the oblique cell wall. Since not all cells show oblique wall, they should show how many cells they did the quantification and what percentage of cells with oblique wall is.

7. In figure 5, they observed the defective cell wall in mutants with disrupted BR signaling. They thus made the statement that optimal BR signaling is the key to maintain the correct cell wall replacement. I think this part is a little too preliminary. It would be nice if they perform the experiment with dose gradient of BR signaling.

8. In the background of bri1-116 and bri-triple mutants, they found that the expression of BRI1 in endodermis (pSCR-BRI1) or stele (pSHR-BRI1) largely rescued cell wall orientation in the cortex of bri1-116 and bri-triple.

However, the expression of BRI1 in non-hair cells (pGL2-BRI1) exacerbated the phenotype of cell wall orientation, which is confusing. Have they tried pWER promoter? How can they explain this opposite observations?

9. The authors showed that the position of the YFP-POK1-labeled CDZ was often consistent with PPB in WT, but not in PMEIox roots. If this can also be observed in Bri1 mutants or BR treated roots? Another question I have is how could they determine the cell with PPB in figure 6B was defective? As shown in previous images, many cells in PMEIox roots maintained normal cell wall orientation. I just wonder how they could decide which cell is defective before the final cell plate is formed in the cytokinesis. If this is impossible, how the quantification in this figure was made. This is important because it decides if the conclusion in this part is valid or not.

© 2021. Published by The Company of Biologists under the terms of the Creative Commons Attribution License (https://creativecommons.org/licenses/by/4.0/).

Minor issues:
1. Figure 1, cnu1 should be labeled as cnu1/ PMEIox if that is the crossed line.
2. Figure 2E, I saw the fungal contamination in the growth medium. If that is the case, they need to repeat the experiments as the fungal contamination can greatly affect the root growth direction.
3. The manuscript needs language editing. There are many grammatical errors. In addition, the logic flow in many parts of introduction is ambiguous and hard to follow. A few statements in the abstract is also hard to understand.

Reviewer 3

Advance summary and potential significance to field

In this manuscript the authors investigate the role of BR signalling in cell wall orientation in the Arabidopsis root meristem.
They show that inhibition of pectin methiltransferase mediated by PMEI5 induces BR signalling, via RLP44 and BRI1, and results in oblique cell wall phenotype. The authors concluded that cell wall signalling-triggered elevation of BR signalling is causative for the oblique cell wall phenotype in PMEIox plants.

They then performed tissues specific PMEI5 expression to separate the different phenotype: root waving, root growth inhibition, and aberrant cell wall orientation and demonstrate that root waving is independent from meristematic cell shape defects because when PMEI5 is expressed in the root hair cell file or in the xylem, roots display waving defect but not aberrant wall orientation defects. When PMEI5 was expressed in the cortex aberrant wall orientation defects where observed but only in this tissue.

By analysing the root meristem of br1-1 mutants where BR signalling is strongly reduced they found the same aberrant wall orientation defects observed in plant overexpressing PMEI5 where BR signalling is enhanced.

They that show that cell wall perturbation induced by PMEI5 leads to cytokinesis defects after the CDZ is specified. The role of BR signalling in this process is not investigated.

Comments for the author

This manuscript has several interesting elements but at times is very confusing.
How can the authors conclude that elevation of BR signalling is needed for the oblique cell wall phenotype in PMEIox plants only to show after that in BR signalling mutant (where BR signalling is virtually absent) you have exactly the same phenotype? I suspect that the phenotypes are not the same and that probably the role of BR in this process is very marginal.
Probably to better understand the role of BR in this process the authors should perform the same experiments performed in PMEIox, to asses whether the aberrant cell wall placement observed in this mutants is associated with defects in the cell division plane orientation, in the bri1 and the bri-triple, mutants.

First revision

Author response to reviewers' comments

>>We thank all three reviewers for their interest in our work and their constructive criticism. We have addressed all comments in a point-by-point response below. Our revised manuscript now contains additional experiments and extensive re-analysis (quantification) of our data. In addition, the text was edited significantly to improve readability. We hope the reviewers agree with our view that the revised manuscript is strongly improved and find it acceptable for publication.

© 2021. Published by The Company of Biologists under the terms of the Creative Commons Attribution License (https://creativecommons.org/licenses/by/4.0/).

Reviewer 1 Advance Summary and Potential Significance to Field:
In the manuscript by Li and co-workers, the authors connect BR signaling and cell wall orientation in the model species Arabidopsis.

Reviewer 1 Comments for the Author:
Besides the scientific remarks indicated below, the manuscript is kind of hard to read due to unnecessary long and complicated sentences. I would advise a serious rework to make the text more easy to read.

>>Following the reviewer's advice, we have thoroughly edited the manuscript to improve its readability.

Additionally, figures are not of the highest quality (might be a pdf conversion problem)

>>We assume this indeed to be a problem of the initial submission or PDF conversion. We now provide higher resolution figures, which should fix this issue.

and there is a general sloppiness (not being precise) in the manuscript and figures. For example the text is full of non-scientific expressions such as "slightly", "mild" etc. Results are either significant if proper statistical testing is done; or they are not.

>>The manuscript has been edited significantly according to the reviewer's suggestion.

In one of the figures (Fig2E) dealing with root waving, there is what seems to be a bacterial contamination on the plate and the root closest to this shows a wave effect, while the other do not. Obviously this makes any interpretation hard and gives the impression this experiment was only done once. It that respect, how many times were these experiments repeated?

>>We apologize for this. However, we would like to note that plants were placed on this plate solely for imaging, after being grown separately on DMSO and DEX plates, respectively. The experiment was done several times with identical results in various generations of the transgenic lines. In this new version of the manuscript, we provide quantification using the vertical growth index derived from the original plates and only show the images of the waving lines.

Related to this, there is no indication how often the oblique divisions occur in different lines/conditions, or how many roots/experiments each image is representative of in Fig1-4. This should be clearly mentioned and should include a number of samples large enough to do proper statistical testing. This info must be added and the frequency of oblique divisions in different tissues, per root or per. e.g. 100 cells of the given type, should also be included, to assess the strength of the phenotypes, similarly to what is done in Fig5. Now the *oblique divisions in Fig1-4 are presented as a binary phenotype (oblique divisions yes/no), while for example, the phenotype of constitutive PMEIOX (Fig1C) and the conditional one (3A) are very different in strength.*

>>Following the reviewer's suggestion, we now provide quantification for Figures 1-4, including statistical testing.

Could the authors additionally give an explanation for this?

>>After quantification, both root waving and oblique cell division phenotypes appear similar in strength between PMEIox and pUBQ10>GR>PMEI5. However, it is conceivable that the two promoters used (pUB10 in the inducible line, p35S in the constitutive line) might be responsible for some variation based on their relative strength in different tissues. For example, based on other transgenic fluorescent lines available in the lab for other projects, the relative strength of the 35S promoter seems higher in the stele, whereas the strength of the UBQ10 promoter seems more uniform. This hypothesis is particularly tempting in light of the various non-cell autonomous effects observed with BR signalling and PMEI5 expression. Based on QRT-PCR (Fig S3) the overall expression is higher in the inducible line. However, in the absence of tissue-specific expression data, we would rather refrain from speculating about potential (small) differences between the phenotypes of the two lines.

© 2021. Published by The Company of Biologists under the terms of the Creative Commons Attribution License (https://creativecommons.org/licenses/by/4.0/).

Also related to this general remark, some statistics: t-test (Fig.S1A, S4) and ANOVA (5A) are reported. These are only valid for normally distributed data – has the normality of distribution been tested? If data is not normal, other types of tests must be applied (Wilcoxon, Kruskal-Wallis..). Can the authors please evaluate this for all their experiments and data? Also, the significance of categorical data (Fig.5B,C and similar quantifications from other experiments – see above) should also be tested by e.g. the CHI-squared test.

>>We thank the reviewer for this suggestion and have assessed normality using Shapiro-Wilk test. In several cases, we have re-analyzed the data accordingly, using Kruskal-Wallis, followed by Dunn's test.

On line 316-318, to optimally support the statement made, time-lapse imaging of PMEIOx plants expressing markers of both POK1 and MTs would have to be performed (e.g. in an F1 cross of PMEI-OX with YFP-POK1; RFP-MBD (Lipka et al., 2014). These lines are published and making the cross with analysis in F1 would be feasible. Currently, the statement is very bold based on the available data.

>>We agree that this would be a very valuable experiment and, following the reviewer's suggestion, have generated this biological material. Unfortunately, imaging of the YFP-POK1 line is very challenging with the equipment available to us, and we were unable to perform time lapse experiments due to bleaching and to the relative scarcity of aberrant cell division events, even in PMEIox. Consequently, to avoid misinterpretations, we have toned down the conclusions accordingly.

For the tissue specific complementation experiments, there is no control to validate these lines are functional.
E.g. Fig2F shows no effect by inducing PMEI5 in the endodermis, but there is no way of knowing this line is functional, so we could be looking at WT plants. Did the authors include a GFP tag in these lines to verify they are properly expressing the construct. Alternatively, did the authors validate the induction of the lines via Q- RT-PCR or an equivalent method? Given the importance given to these results, these lines would need to be validated before using.

>> Initially, we had generated several lines for each construct and performed the experiments with the highest expressing line in each case (based on QRT-PCR). We have now added QRT-PCR analysis of PMEI5 expression in the transgenic lines used as Figure S3. All lines show several fold induction compared to the uninduced control. The absolute expression levels expectedly vary greatly between the lines, presumably at least in part due to the spatial specificity of the promoters. Nevertheless, we have toned down the conclusion regarding lines that do not show any root phenotype. It is maybe of interest here that both pGL2>GR>PMEI5 and pRLP44>GR>PMEI5 show a distinct above-ground phenotype (see included Figure, E, F), suggesting that PMEI5 is functional and expressed at high enough levels to elicit an altered morphological phenotype in the shoot (see Figure for Reviewers 1 below). We did not include these data in the manuscript, as we think the above- ground morphology is out of scope of the work presented here. We would also like to note that dismissing all lines that do not show any phenotype in roots, i.e. pGL2>GR>PMEIox, pCASP1>GR>PMEIox, and pRLP44>GR>PMEIox, would not alter our conclusions regarding differential behaviour of the pCOBL9 and pCO2 lines.

© 2021. Published by The Company of Biologists under the terms of the Creative Commons Attribution License (https://creativecommons.org/licenses/by/4.0/).

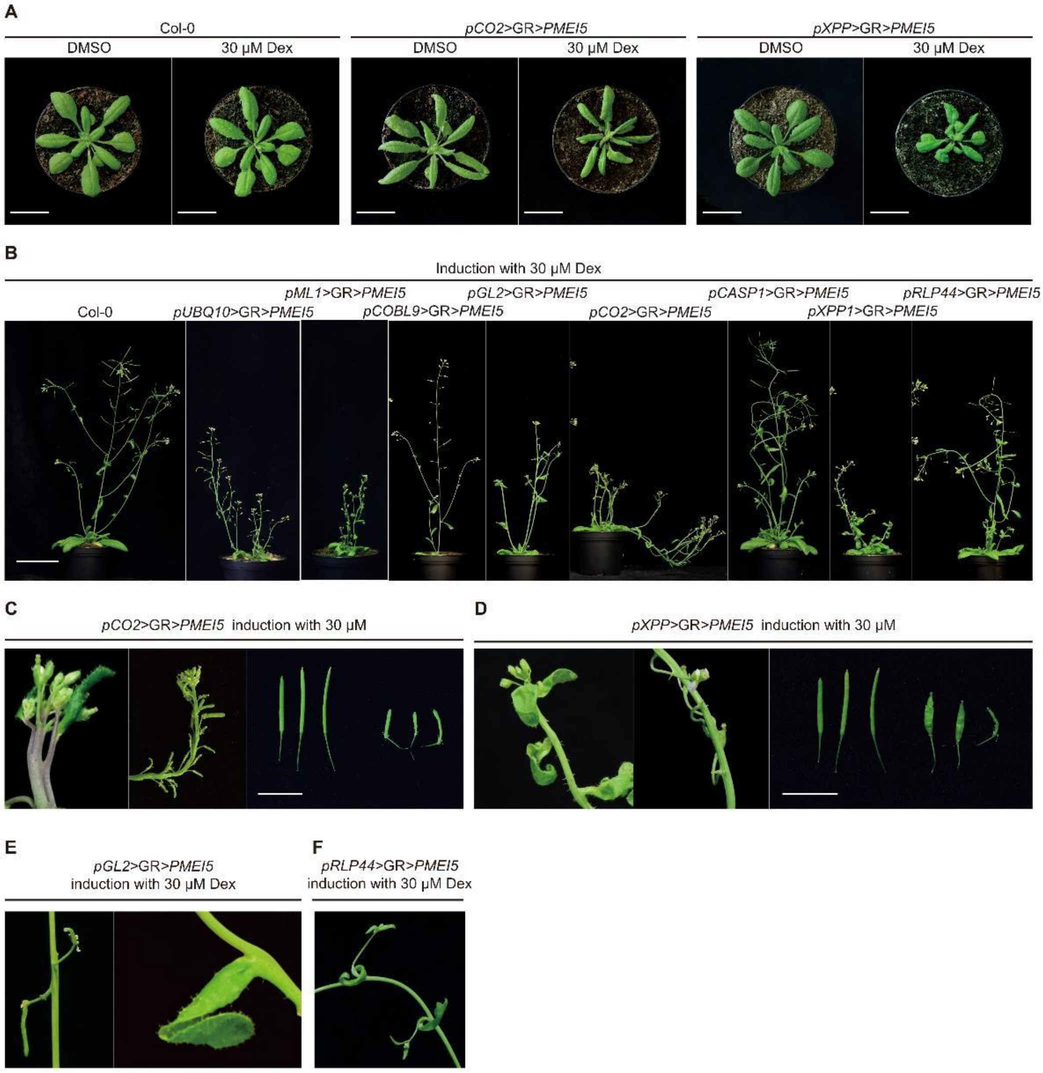

Figure for Reviewers 1. Above-ground phenotype of tissue specific expression lines. Please note phenotype of pGL2>GR>PMEI5 (E) and pRLP44>GR>PMEI5 (F). These lines do not show root waving, nor cell wall orientation defects in the RAM (Fig 2 and 3 in the MS). Taken form Li, Zhenni, PhD thesis, Heidelberg University.

Lines 294-299: formally the argument about alignment of cell division with the PPB cannot be made if the two are not analyzed in the same cells. Moreover, time-laps analysis with quantification would also make a stronger point. The authors should consider making the cell biological data more convincing as suggested or alternatively reduce the strength of their claims and replace PPB position by CDZ or similar.

>>Due to the challenges faced with imaging of POK1 (see also above), we were not successful in performing this experiment. Accordingly, we have cautiously toned down our conclusions as suggested by the reviewer.

Line 299 implies that cell plate positioning is defective in ALL PMEIOx cells, which is not true – please reword.

>>We have rephrased this sentence according to the reviewer's suggestion.

Smaller remarks that need to be addressed:
Fig.5C, bri1-116 pSHR::BRI1 – n=3 is not acceptable; increase the sample size or remove

© 2021. Published by The Company of Biologists under the terms of the Creative Commons Attribution License (https://creativecommons.org/licenses/by/4.0/).

>>We have removed these data.

Fig.5D pSHR-BRI: some roots show rescue and others do not. Can the authors explain this?

>> Root length shows high variability, also in WT (this is reflected in the large range in values in Fig. 5E, and please note the short WT root in the third seedling from left, Fig. 5D). In addition to this variability, the levels of BRI1 driven by the SHR promoter could potentially vary among seedlings and with time after germination. In any case, the quantifications of root length and cell wall orientation were performed on these same conditions, demonstrating no linkage between the two.

Line 51-52: is this precise?

>> We agree that different definitions of asymmetric cell division are in use, therefore this sentence might be confusing; consequently, we have opted for removing it.

Fig.1: what does the asterisk mean? Not in the legend.

>>We have removed the asterisk.

Also, no mentioning of WT grown on BL in the text (panel A).

>>We have now added a sentence referring to this.

Moreover, there is no correlation between the root waving phenotypes in panel A and the cellular phenotypes in panels B-E.

>>As we have now quantified all the phenotypes, this should make comparisons easier. However, uncoupling of the root waving and cellular phenotype is also observed with, for example, the pCO2>GR>PMEI5 line. We therefore conclude that oblique cell walls are not causal for root waving.

Next, line 143 (Legend Fig 1) – some strange typo.

>> We thank the reviewer for spotting this; the sentence is now corrected.

Also, preferably show 3-4 seedlings per genotype as they grew on the plate

>> Please note that this figure panel (A) is only for illustrative purposes; the root waving and suppression phenotypes are extensively quantified in our previous work (Wolf et al., 2012, 2014) and in Lanza et al., 2012.

Line 150: why only complementation of cnu2, but not cnu1, at this moment, even though the conclusion at the end of the paragraph covers both BRI and RLP44.

>>Complementation of cnu1 is not straightforward, because the mutation is hypomorphic. Additional BRI1 expression, even under the native promoter, causes severe overexpression effects. However, other data now included in the manuscript, namely BL-induced cell wall orientation defects (Fig. 6) and suppression of the pCO2>GR>PMEI5 cell wall orientation phenotype by an independent (hypomorphic) bri1 allele, bri1^{cnu4} (Fig. S7), further support our conclusion.

Line 180: yes it is waving, but in a way very different from the other lines
>> We have amended the sentence so that it does not imply that both root waving phenotypes are necessarily identical.

Line 185-186: This statement is likely correct in the context of your experiment, but the statement you make here is rather general. Please rephrase to make sure you are not generalizing your conclusions throughout the text.

© 2021. Published by The Company of Biologists under the terms of the Creative Commons Attribution License (https://creativecommons.org/licenses/by/4.0/).

>>We thank the reviewer for this suggestion; we have rephrased the conclusions throughout the text to make clear they only refer to the PMEI/BL context.

Fig.3: DEX in B, mock in D are very bad images, as if chloral hydrate diffused into the water immersion. The quality of these images should be improved.

>> We have now replaced these panels with higher quality images.

Lines 195-196: pCO2 is reported not to be entirely specific to cortex, so for claims of non-cell autonomous action its expression domain should be analyzed closely, or at least the possibility of leaky expression should be mentioned.

>>We now show an additional Supplementary Figure (Fig S4) showing that in the pCO2 driver line the trans- activation seems to be indeed restricted to the cortex cells. However, we now also state that unspecific expression cannot be formally excluded. In addition to this caveat, PMEI5 itself might be mobile in the wall, as stated in the manuscript.

Also on these lines, the phenotypes are described for specific cell files, although I also see defects in others. This all shows this phenotype needs to be much more closely evaluated and quantified to allow these statements to be made.

>> We have now added separate quantifications for cortex and epidermis (Fig 4 C,E), with similar results. Endodermis cell wall orientation was much more difficult to quantify (due to the shape of endodermal cells), so we have refrained from showing these data in this manuscript.

Figure4: Root meristems only reach their steady state growth rate around 5-6 days after germination, yet growth experiments were performed on very young plants: e.g. 2days. What is the impact of analyzing such young plants for growth defects?

>>We believe this to be a misunderstanding. All roots are imaged at 5DAG; the numbers indicate the day when the roots were transferred to induction medium. Accordingly, the cell wall orientation defects are more severe in roots transferred at 2DAG than in those transferred at 4DAG.

Also in Fig4 and others, the resolution/magnification of the figures is not good enough to evaluate the phenotypes described.

>>We have increased the quality of the figure panels and added the quantification (panels C and D) to fix this problem.

Line 211-212: What does “an oblique newly wall orientation defects” mean? Please revise this sentence.

>> This mistake is now corrected.

Same for Line 214-216: I do not understand these sentences.

>>We have rephrased these sentences, for clarity.

Line 242: why spatiotemporal, where is the temporal effect?

>>We have now rephrased this sentence.

Line 257: you can only talk about correlation if you do statistical testing. Do you mean perhaps uncoupled or unlinked?

>>We have amended the sentence according to the reviewer’s suggestion.

Fig6: these observations are done in different cell files, while the authors have shown there can be

© 2021. Published by The Company of Biologists under the terms of the Creative Commons Attribution License (https://creativecommons.org/licenses/by/4.0/).

a difference in response between them. Did the authors take this into account?

>>The reviewer is correct in that both epidermal and cortex cells were used for this analysis. However, we think cell type is unlikely to play a big role here, given the rather homogenous effects in the ubiquitous PMEIox line. We have added the information that analysed cells are not derived from a single tissue to the text.

Figure S5 is difficult to read. it would be helpful to display the meristem size as bars in a different color and a scale on a secondary Y-axis. Significance of the data (oblique divisions per 10 cells) should be tested by CHI- squared test as outlined in the major comments

>>We have re-organized the figure as suggested by the reviewer.

Reviewer 2 Advance Summary and Potential Significance to Field:
This manuscript by Li et al. reported the involvement of BR signaling in cell wall orientation in Arabidopsis roots. Cell division orientation is one of the key mechanisms that regulate tissue patterning during development. This is particularly important during plant organogenesis as all plant cells are encased in rigid cells. Previous studies from the same lab have shown that *impaired physical properties of the cell wall promote BR signaling. They found that overexpression of pectin methylesterase inhibitor protein 5 (PMEI5) could activate BR signaling through RLP44, resulting in developmental phenotypes including organ fusion and root waving. In this paper, Li et al. made efforts to further connect the oblique cell division found in PMEI5 overexpression lines to BR signaling. This is a very interesting observation and most experiments were nicely designed and well performed. However, I think substantial revision is required to make it a publication at Development. My major concerns are as follows:*

Reviewer 2 Comments for the Author: Major concerns:

1. In figure 1, they showed altered cell wall orientation in PMEIox roots was largely restored in mutants with lesions in BRI1 (cnu1) and RLP44 (cnu2). Since not all cells are affected, quantitative analysis of oblique cell wall with proper statistics is needed here to evaluate the extent of cell division phenotypes in different backgrounds.

>>Following the reviewer's advice, we have now added quantifications with statistics to all figures showing cell wall orientation phenotypes in cortex cells, epidermal cells, or both.

In addition, if the elevated BR signaling *is the key translator of the cell wall defects in cell division, they should also be able to find oblique cell wall in brassinolide treated roots.*

>> This experiment is now included as Figure 6 in the revised manuscript. Indeed, both reduced and increased BR signalling leads to oblique cell wall placement.

2. In the title and also many other places in the paper, the authors claimed that optimal BR signaling is important. However, I did not find any data showing the examination of BR signaling strength. In Figure 1, have they examined the effect of different concentrations of brassinolide? Can they find the optimal level that BR signaling promotes the cell division defect?

>> We believe Figure 6 largely addresses this point (see previous answer). In addition, we now also include more genetic evidence that the PMEI5-mediated phenotypes described are BR-dependent, because the weak bri mutant bri1^{cnu4} *supresses the pCO2>GR>PMEI5 cell wall orientation phenotype.*

3. In figure 2, they inducibly mis-expressed PMEI5 in different cell types. How can they prove the correct expression of the gene? Why did they add a fluorescent tag to the PMEI5? Or if the disruption of the protein function is s concern, they should presented other evidence showing the successful mis- expression of PMEI5.

>>We now include QRT-PCR data showing induction of PMEI5 in all lines compared to the respective controls. Please also see the response to Reviewer 1. We provide a Figure (Figure 1

© 2021. Published by The Company of Biologists under the terms of the Creative Commons Attribution License (https://creativecommons.org/licenses/by/4.0/).

above) showing that also lines without root phenotype display PMEI5-induced growth defects in the shoot, providing circumstantial evidence that PMEI5 is expressed and functional.

4. Among different lines (pUBQ10>GR>PMEI5, pML1>GR>PMEI5, pCOBL9>GR>PMEI5 and pXPP>GR>PMEI5), they only found pUBQ10>GR>PMEI5 roots had both root waving phenotypes and defective cell wall placement. However, BR level should be elevated in all four lines. Have they performed any quantification to dissect if the phenotypes were caused by distinct BR level in different lines, or by different spatial distribution?

>> In the absence of a good cellular BR response marker, we cannot unequivocally show whether the two phenotypes in question are separable by a BR signalling threshold. While that seems unlikely, given that one line (pCOBL9>GR>PMEI5) shows root waving but no cell wall orientation phenotype whereas the reverse is true for another (pCO>GR>PMEI5), we think answering this question is beyond the scope of the current study. New Fig. S7 now also provides independent evidence that the occurrence of the cell wall orientation defect in the absence of root waving (pCO2>GR>PMEI5) is dependent on BRI1/BR signalling.

5. Interestingly, when specifically expressed in cortex (pCO2>GR>PMEI5), misexpressed PMEI led to oblique cell walls without occurrence of root waving. However, the cell wall phenotype seemed to occur in both cortex and epidermis. Could they explain this phenomenon?

>>We cannot provide a comprehensive answer to this important question. Non-cell autonomous effects have been observed extensively with BR signalling, including this manuscript (Fig 5), and it is not clear how these are mediated. Besides possible signalling-mediated cell-cell communication, it seems likely that mechanical feedback between cortex and epidermis and between other cell files exists. However, in the absence of direct evidence, we would rather refrain from speculating. As we mention in the manuscript, we cannot rule out apoplastic movement of PMEI5 either. Unfortunately, since PMEI5 does not tolerate any tag (our previous work), this possibility could not be directly addressed at this point.

In addition, the phenotype appeared not as *dramatic as in PMEIox lines. Is that because of the expression level? Quantification of the cell wall phenotype should be provided.*

>> The expression levels are difficult to compare by QRT-PCR (see Fig. S3) as the CO2 promoter is only active in a few cells of the root. However, the strength of the phenotype is rather similar to that in PMEIox in cortex and epidermis, according to our quantification (Fig 4).

6.I found the variation of oblique phenotypes in the images they *provided. In figure 1c, PMEIox roots had very strong cell wall defects, while pUBQ10>GR>PMEI5 root in figure 3A seemed to have fairly mild phenotype. Again, I think the authors should make it clear that how they evaluate the oblique cell wall. Since not all cells show oblique wall, they should show how many cells they did the quantification and what percentage of cells with oblique wall is.*

>> We now provide quantification of oblique cell walls per root tissue in median confocal sections. According to these data, the cortical cell wall orientation phenotype of pUBQ10>GR>PMEI5 is similar to that of PMEIox.
Differences are however not inconceivable, given that the relative strength of the promoter might differ between tissues.

7. In figure 5, they observed the defective cell wall in mutants with disrupted BR signaling. They thus made the statement that optimal BR signaling is the key to maintain the correct cell wall replacement. I think this part is a little too preliminary. It would be nice if they perform the experiment with dose gradient of BR signaling.

>>We thank the reviewer for this suggestion. We have now performed a BR dose-response experiment (Fig. 6) and indeed observe aberrant cell wall orientation in both BR-deficient (PPZ treated) plants and those treated with elevated exogenous concentrations of BL.

8. In the background of bri1-116 and bri-triple mutants, they found that the expression of BRI1 in

© 2021. Published by The Company of Biologists under the terms of the Creative Commons Attribution License (https://creativecommons.org/licenses/by/4.0/).

endodermis (pSCR-BRI1) or stele (pSHR-BRI1) largely rescued cell wall orientation in the cortex of bri1-116 and bri-triple.
However, the expression of BRI1 in non-hair cells (pGL2-BRI1) exacerbated the phenotype of cell wall orientation, which is confusing. Have they tried pWER promoter? How can they explain this opposite observations?

>> We thanks the reviewer for this comment. The effect of BRI1 when expressed in non-hair cells could be a result of supra-optimal BR signalling intensity (Fridman et al. 2014; Vragovic et al., 2015).

We added the following to the discussion:

"For example, pGL2-BRI1 in bri1-triple has long root meristems and almost wild type-like root length and shows higher an increased number of anticlinal divisions (Hacham et al., 2011, Vragovic et al., 2015), but harbours severe wall orientation defects. This could be the result of high local BR signalling intensity (Fridman et al., 2014, Vragovic et al., 2015), as in pML1>GR>PMEI5. "

9. The authors showed that the position of the YFP-POK1-labeled CDZ was often consistent with PPB in WT, but not in PMEIox roots. If this can also be observed in Bri1 mutants or BR treated roots?

>>We agree this would be an interesting and valuable experiment. Unfortunately, the imaging of POK1 is rather challenging and we were unable to obtain conclusive results, even with pharmacological treatments (PPZ or BL). We have therefore toned down our conclusions.

Another question I have is how *could they determine the cell with PPB in figure 6B was defective? As shown in previous images, many cells in PMEIox roots maintained normal cell wall orientation. I just wonder how they could decide which cell is defective before the final cell plate is formed in the cytokinesis. If this is impossible, how the quantification in this figure was made. This is important because it decides if the conclusion in this part is valid or not.*

>>We believe this to be a misunderstanding. At the pre-prophase stage, it not possible to determine which cell will turn out to be defective, as the distribution of PPB angles is similar in Col-0 and PMEIox (Fig 7I, previously 6I). We quantified the various division plan indicators (PPB (I), metaphase plate (J), chromatid midline (K), cell plate (L)) in random cells, regardless of their phenotype. Our conclusions are based on these respective distributions.

Minor issues:
1. Figure 1, cnu1 should be labeled as cnu1/ PMEIox if that is the crossed line.

>>We apologize for the misunderstanding: this is the EMS-induced bri1 mutation in the PMEIox background initially described in Wolf et al., 2012.

2. Figure 2E, I saw the fungal contamination in the growth medium. If that is the case, they need to repeat the experiments as the fungal contamination can greatly affect the root growth direction.

>> We apologize for this. Please note that plants were placed on this plate solely for imaging, after being grown separately on DMSO and DEX plates, respectively. The experiment was done several times with identical results in various generations of the transgenic lines. In this new version of the manuscript, we provide quantification using the vertical growth index derived from the original plates and only show the images of the waving lines.
This experiment has been repeated more than three times with identical results; we now also provide quantification of the original plates in Figure 2.

3. The manuscript needs language editing. There are many grammatical errors. In addition, the logic flow in many parts of introduction is ambiguous and hard to follow. A few statements in the abstract is also hard to understand.

>>We have revised the manuscript extensively to improve readability and ease understanding.

© 2021. Published by The Company of Biologists under the terms of the Creative Commons Attribution License (https://creativecommons.org/licenses/by/4.0/).

Reviewer 3 Advance Summary and Potential Significance to Field:
In this manuscript the authors investigate the role of BR signalling in cell wall orientation in the Arabidopsis root meristem.
They show that inhibition of pectin methiltransferase mediated by PMEI5 induces BR signalling, via RLP44 and BRI1, and results in oblique cell wall phenotype. The authors concluded that cell wall signalling-triggered elevation of BR signalling is causative for the oblique cell wall phenotype in PMEIox plants.

They then performed tissues specific PMEI5 expression to separate the different phenotype: root waving, root growth inhibition, and aberrant cell wall orientation and demonstrate that root waving is independent from meristematic cell shape defects because when PMEI5 is expressed in the root hair cell file or in the xylem, roots display waving defect but not aberrant wall orientation defects. When PMEI5 was expressed in the cortex aberrant wall orientation defects where observed but only in this tissue.

By analysing the root meristem of br1-1 mutants where BR signalling is strongly reduced they found the same aberrant wall orientation defects observed in plant overexpressing PMEI5 where BR signalling is enhanced.

They that show that cell wall perturbation induced by PMEI5 leads to cytokinesis defects after the CDZ is specified. The role of BR signalling in this process is not investigated.

Reviewer 3 Comments for the Author:

This manuscript has several interesting elements but at times is very confusing.
How can the authors conclude that elevation of BR signalling is needed for the oblique cell wall phenotype in PMEIox plants only to show after that in BR signalling mutant (where BR signalling is virtually absent) you have exactly the same phenotype? I suspect that the phenotypes are not the same and that probably the role of BR in this process is very marginal.

>> Based on this and our previous work, we have to assume all the PMEIox phenotypes (except for some primary effects that are not relevant here), including the oblique cell wall phenotype, are caused by BR signalling activation, mediated by RLP44 (Wolf et al., 2012; Wolf et al., 2014; Holzwart et al., 2018; Holzwart et al., 2020). In the revised manuscript, we provide additional evidence as the PMEI5-induced phenotypes in the pCO2>GR>PMEI5 line are suppressed by a hypomorphic mutation in BRI1 (Fig. S7). Moreover, Fig. 6 in the revised manuscript now shows that oblique cell wall orientation phenotype occurs in BR-depleted plants as well as in plants with enhanced BR signalling due to external application of BL.

Probably to better understand the role of BR in this process the authors should perform the same experiments performed in PMEIox, to asses whether the aberrant cell wall placement observed in this mutants is associated with defects in the cell division plane orientation, in the bri1 and the bri-triple, mutants.

>> We agree that this would be an experiment (see also Reviewer 1's comment above). Unfortunately, imaging of the YFP-POK1 line is very challenging with the equipment available to us, and the number of fusion events with sufficient specific YFP-POK1 fluorescence in plants with reduced BR signalling was too low. To avoid misinterpretations, we have toned down the conclusions from the PMEIox experiment. However, we feel that new Figures 6 and S7 strongly support our conclusions regarding the involvement of BR signalling in the control of cell wall orientation.

© 2021. Published by The Company of Biologists under the terms of the Creative Commons Attribution License (https://creativecommons.org/licenses/by/4.0/).

Second decision letter

MS ID#: DEVELOP/2021/199504

MS TITLE: Optimal BR signalling is required for adequate cell wall orientation in the Arabidopsis root meristem

AUTHORS: Zhenni Li, Ayala Sela, Yulia Fridman, Lucia Garstka, Herman Hofte, Sigal Savaldi-Goldstein, and Sebastian Wolf

I have now received all the referees reports on the above manuscript, and have reached a decision. The referees' comments are appended below, or you can access them online: please go to BenchPress and click on the 'Manuscripts with Decisions' queue in the Author Area.

The overall evaluation is positive and we would like to publish a revised manuscript in Development, provided that the referees' comments can be satisfactorily addressed. Please attend to all of the reviewers' comments in your revised manuscript and detail them in your point-by-point response. If you do not agree with any of their criticisms or suggestions explain clearly why this is so.

We are aware that you may currently be unable to access the lab to undertake experimental revisions. If it would be helpful, we encourage you to contact us to discuss your revision in greater detail. Please send us a point-by-point response indicating where you are able to address concerns raised (either experimentally or by changes to the text) and where you will not be able to do so within the normal timeframe of a revision. We will then provide further guidance. Please also note that we are happy to extend revision timeframes as necessary.

Reviewer 1

Advance summary and potential significance to field

The authors have addressed most of my remarks. A few smaller remaining issues are listed below.

Comments for the author

- quantification is now convincing, but I did not find the # of independent repeats of each experiment except the vague statement in the response to reviewers
- sample sizes are generally sufficient, but in Figs 4 and 6 there are samples with n=5, which is really borderline, especially with no info on the # of repeats of the experiment.
- Fig. S3 would greatly benefit from using a logarithmic scale on the Y-axis
- The quality of the DEX image in Fig. 3B is somewhat better than the previous version, but still quite poor

Reviewer 2

Advance summary and potential significance to field

The report of the involvement of BR signaling in cell wall orientation can help us to understand the mechanisms regulating tissue patterning during plant development.

Comments for the author

The authors have addressed all my concerns in the last review by adding additional results or providing in-depth discussions. I am satisfied with the current version and would like to recommend the publication.

© 2021. Published by The Company of Biologists under the terms of the Creative Commons Attribution License (https://creativecommons.org/licenses/by/4.0/).

Reviewer 3

Advance summary and potential significance to field

This revised version of the manuscript is certainly improved.

Comments for the author

Nevertheless the role of BR in this process is still confusing to me probably due, as the authors also admit, to the luck of suitable tools. I still believe that to draw conclusion it is important to perform the same experiments performed in PMEIox, to asses whether the aberrant cell wall placement observed in this mutants is associated with defects in the cell division plane orientation, in the bri1 and the bri-triple, mutants.

Second revision

Author response to reviewers' comments

Reviewer 1 Advance Summary and Potential Significance to Field:
The authors have addressed most of my remarks. A few smaller remaining issues are listed below.

Reviewer 1 Comments for the Author:
- quantification is now convincing, but I did not find the # of independent repeats of each experiment except the vague statement in the response to reviewers

>>*We apologize for this omission; all experiments have been repeated at least twice, which is now indicated in the Material and Methods section.*

- sample sizes are generally sufficient, but in Figs 4 and 6 there are samples with n=5, which is really borderline, especially with no info on the # of repeats of the experiment.

>> *These experiments have ben repeated at least twice, with identical results. We therefore kept the figure as it is, but added a note indicating the repeats to the Material and Methods section.*

- Fig. S3 would greatly benefit from using a logarithmic scale on the Y-axis

>> *We thank the reviewer for this suggestion and have updated Figure S3 accordingly.*

- The quality of the DEX image in Fig. 3B is somewhat better than the previous version, but still quite poor

>> *We apologize for the suboptimal quality of the image. Unfortunately, images from the repetitions of this experiment used a different staining method and would not fit to the other figure panels. Due to shortage of personnel and restricted microscope access in these challenging times, we have been unable to conduct a new experiment to improve image equality and hope the reviewer can accept this decision.*

Reviewer 2 Advance Summary and Potential Significance to Field:
The report of the involvement of BR signaling in cell wall orientation can help us to understand the mechanisms regulating tissue patterning during plant development.

Reviewer 2 Comments for the Author:
The authors have addressed all my concerns in the last review by adding additional results or providing in-depth discussions. I am satisfied with the current version and would like to recommend the publication.

>>*We thank the reviewer for the appreciation of our efforts.*

© 2021. Published by The Company of Biologists under the terms of the Creative Commons Attribution License (https://creativecommons.org/licenses/by/4.0/).

Reviewer 3 Advance Summary and Potential Significance to Field:

This revised version of the manuscript is certainly improved.

Reviewer 3 Comments for the Author:
Nevertheless the role of BR in this process is still confusing to me probably due, as the authors also admit, to the luck of suitable tools. I still believe that to draw conclusion it is important to perform the same experiments performed in PMEIox, to asses whether the aberrant cell wall placement observed in this mutants is associated with defects in the cell division plane orientation, in the bri1 and the bri-triple, mutants.

>>We respectfully disagree with the reviewer. We show that BR signalling is required for cell wall orientation (during cytokinesis), as both BR deficient mutants and BR treatment or signalling overactivation leads to mis- aligned anticlinal walls. In the PMEIox background, which shows an activation of BR signalling to compensate for its cell wall defect, our experiments (Fig 7) suggest that the fusion site of the expanding cell plate is still marked by POK1, suggesting that guidance by the cortical division zone (CDZ) might still be operational, but that the position of the CDZ might be shifted. Due to the low number of oblique events in the bri1 mutant, the experiment suggested by the reviewer would have to be performed in the triple mutant background; generating these results would be extremely challenging, labour-intensive, and time-consuming, and is not feasible in the present conditions of limited personnel and restricted microscope access. Please note that we do not claim that defective cell wall orientation in BR deficient mutants occurs through the same mechanisms as in plants with supra-optimal BR signalling. On the other hand, we have provided compelling evidence that both reduced and increased BR levels cause a cell wall orientation defect, as the reviewer requested. We do not attempt to provide mechanistic insight into this defect in the current manuscript, as this is would require investigating more markers in addition to POK1; we believe this is out of the scope of this study, but will be investigated in future work.

Third decision letter

MS ID#: DEVELOP/2021/199504

MS TITLE: Optimal BR signalling is required for adequate cell wall orientation in the Arabidopsis root meristem

AUTHORS: Zhenni Li, Ayala Sela, Yulia Fridman, Lucia Garstka, Herman Hofte, Sigal Savaldi-Goldstein, and Sebastian Wolf
ARTICLE TYPE: Research Article

I am happy to tell you that your manuscript has been accepted for publication in Development, pending our standard ethics checks.

Reviewer 2

Advance summary and potential significance to field

This study provides evidence supporting the involvement of BR signaling in the assymetric cell division in arabidopsis roots.

Comments for the author

I have no further question and I think the revised manuscript is ready for a publication now.

© 2021. Published by The Company of Biologists under the terms of the Creative Commons Attribution License (https://creativecommons.org/licenses/by/4.0/).

Reviewer 3

Advance summary and potential significance to field

The manuscript has improved

Comments for the author

I agree with the authors that the experiment I suggest is time consuming and partially out of the scope of this manuscript. I think they should state in the discussion that the role of BR in controlling cell wall orientation is still unclear and that cell wall orientation in BR deficient mutants might occurs through different mechanisms as in plants with supra-optimal BR signalling.

© 2021. Published by The Company of Biologists under the terms of the Creative Commons Attribution License (https://creativecommons.org/licenses/by/4.0/).
